# Social determinants of partner and non-partner sexual violence against women in Chile: a population-based multilevel analysis

**DOI:** 10.1016/j.lana.2026.101656

**Published:** 2026-10-08

**Authors:** Francisca Crispi, Alejandra Fuentes-García, Nicolás Libuy

**Affiliations:** a Escuela de Salud Pública, Universidad de Chile, Independencia 939, Independencia, Santiago, 8380453, Chile; b Escuela de Gobierno, Universidad Adolfo Ibáñez, Diagonal Las Torres 2640, Peñalolén, Santiago, Chile; c Center for Cancer Prevention and Control (CECAN), Santiago, Chile

**Keywords:** Sexual violence, Intimate partner violence, Social determinants of health, Multilevel analysis, Women's health, Chile

## Abstract

**Background:**

Sexual violence against women is a major public health problem in Latin America, yet evidence on its social determinants is scarce and often conflates partner and non-partner forms. We characterised determinants of intimate partner sexual violence (IPSV), non-partner sexual assault (NPSA), and non-partner sexual harassment (NPSH) in Chile and compared their risk profiles.

**Methods:**

We analysed pooled 2017 and 2020 rounds of the Chilean National Survey on Violence in the Family and Other Settings, representative of urban women aged 15 years or older; fieldwork preceded Chile's first COVID-19 case. We fitted multilevel logistic regression for past-year outcomes, women nested in municipalities.

**Findings:**

The sample comprised 13,128 women (8335 for IPSV models). Past-year prevalence was 2.8% (95% confidence interval [CI] 2.4–3.3) for IPSV, 2.5% (2.1–3.0) for NPSA, and 11.3% (10.4–12.3) for NPSH. IPSV was associated with relational factors: dominant relationship (adjusted odds ratio [OR] 6.40, 4.68–8.76) and partner violence elsewhere (2.79, 1.75–4.43); between-municipality variance was negligible. NPSA and NPSH concentrated in younger women and the Metropolitan Region (OR 2.92 [2.00–4.28] and 2.29 [1.77–2.96]); both were associated with social participation (1.49 [1.11–2.00] and 1.26 [1.09–1.46]). Childhood sexual abuse was associated with all three (OR 2.05 [1.45–2.89], 3.03 [2.20–4.20], and 2.06 [1.72–2.46]). Reported non-partner victimisation rose between 2017 and 2020, pre-pandemic.

**Interpretation:**

Risk profiles differed: relational and life-course for IPSV, age-graded and metropolitan for NPSA and NPSH; childhood sexual abuse was common to all three, supporting trauma-informed responses.

**Funding:**

10.13039/501100020884Agencia Nacional de Investigación y Desarrollo, Chile (Doctoral Scholarship 21212141).

## Resumen en español

**Antecedentes:**

La violencia sexual contra las mujeres es un problema de salud pública relevante en América Latina; sin embargo, la evidencia sobre sus determinantes sociales es escasa y combina las formas ejercidas por la pareja con las ejercidas fuera de ella. Caracterizamos los determinantes de la violencia sexual por parte de la pareja íntima (IPSV, por su sigla en inglés), la agresión sexual fuera de la pareja (NPSA) y el acoso sexual fuera de la pareja (NPSH) en Chile, y evaluamos si sus perfiles de riesgo difieren.

**Métodos:**

Analizamos de forma conjunta las rondas 2017 y 2020 de la Encuesta Nacional de Violencia Intrafamiliar y en Otros Espacios de Chile, representativa de las mujeres de 15 años o más residentes en zonas urbanas; el trabajo de campo concluyó antes del primer caso de COVID-19 en Chile. Ajustamos modelos de regresión logística multinivel para los desenlaces de los últimos 12 meses, con las mujeres anidadas en comunas.

**Hallazgos:**

La muestra conjunta incluyó 13 128 mujeres (8335 en los modelos de IPSV). La prevalencia en los últimos 12 meses para IPSV fue de 2,8% (intervalo de confianza [IC] 95% 2,4–3,3), para NPSA 2,5% (2,1–3,0) y 11,3% (10,4–12,3) para NPSH. La IPSV se asoció con factores relacionales: relación dominante (odds ratio ajustado [OR] 6,40; 4,68–8,76) y pareja violenta en otros ámbitos (2,79; 1,75–4,43); la varianza entre comunas fue insignificante. NPSA y NPSH se concentraron en las mujeres más jóvenes y en la Región Metropolitana (2,92 [2,00–4,28] y 2,29 [1,77–2,96]); ambas se asociaron con la participación social (1,49 [1,11–2,00] y 1,26 [1,09–1,46]). El abuso sexual en la infancia se asoció con las tres formas (2,05 [1,45–2,89]; 3,03 [2,20–4,20] y 2,06 [1,72–2,46]). La victimización reportada fuera de la pareja aumentó entre 2017 y 2020, antes de la pandemia.

**Interpretación:**

Los perfiles de riesgo difirieron: relacionales y de curso de vida para la IPSV; asociados a la edad y a la Región Metropolitana para NPSA y NPSH. El abuso sexual en la infancia fue común a las tres formas, lo que respalda respuestas con enfoque de trauma.

**Financiamiento:**

Agencia Nacional de Investigación y Desarrollo (ANID), Chile (Beca de Doctorado Nacional 21212141).

**Research in context**

**Evidence before this study**

We searched PubMed, LILACS, and SciELO for population-based, multilevel analyses of sexual violence against women, published in English or Spanish up to Dec 31, 2025, with no start-date restriction, using terms including “sexual violence”, “intimate partner sexual violence”, “non-partner sexual violence”, “sexual harassment”, and “multilevel”. Most come from sub-Saharan Africa and South Asia, focus on intimate partner violence, and rarely separate sexual from physical violence or partner from non-partner perpetration. Latin American evidence is dominated by Demographic and Health Surveys on intimate partner violence; analyses distinguishing non-partner sexual assault from sexual harassment, with municipal-level random effects, are limited.

**Added value of this study**

We identified no previous population-based multilevel analysis in Latin America that contrasts intimate partner sexual violence (IPSV), non-partner sexual assault (NPSA) and non-partner sexual harassment (NPSH) within a single framework. The three forms show qualitatively different risk profiles: IPSV is dominated by relational dynamics and individual vulnerabilities, with no detectable between-municipality clustering; NPSA and NPSH share a steep age gradient and concentration in the Metropolitan Region. Gender attitudes were inversely associated with non-contact harassment but not with sexual assault, which suggests that contact acts are less subject to differential labelling; both non-partner forms share patterns consistent with differential exposure. Reported non-partner victimisation rose between 2017 and 2020, before the pandemic, coinciding with unprecedented feminist mobilisation in Chile.

**Implications of all the available evidence**

Prevention and clinical responses should treat IPSV, NPSA, and NPSH as distinct phenomena, while recognising childhood sexual abuse as a shared upstream determinant whose prevention could reduce all three. Health and social services should screen for controlling partner behaviours and treat prior childhood sexual abuse as a marker for proactive support. Chile's explicit health guarantee for sexual violence (Garantías Explícitas en Salud, GES 86) can be sharpened by prioritising these high-risk profiles; territorial prevention and gender-equality programming in workplaces and schools remain pending for non-contact harassment, outside GES 86 scope.

## Introduction

Gender-based violence reflects asymmetric power relations between men and women and perpetuates the subordination of women.^1^ Sexual violence, one of its most common forms, is defined by the World Health Organisation (WHO) as any sexual act, attempted sexual act, unwanted sexual comments or advances, or actions to commercialise or exploit a person's sexuality through coercion, irrespective of the relationship to the victim and the setting.^2^ The literature consistently distinguishes three forms with distinct legal, clinical, and epidemiological profiles: intimate partner sexual violence (IPSV); non-partner sexual assault (NPSA), involving forced physical sexual contact by someone other than the partner; and non-partner sexual harassment (NPSH), involving non-contact sexual conduct by someone other than the partner, such as verbal advances, intimidation, and quid-pro-quo propositions.^3^, ^4^, ^5^ These forms share roots in patriarchal power structures and form part of a continuum of sexual violence,^6^ but differ in prevalence, perpetrator profile, and policy framework: in Chile, the explicit health guarantee scheme (Garantías Explícitas en Salud, GES) for sexual violence (GES 86) covers acute clinical care for sexual assault but not for non-contact harassment. At its most severe, this continuum can escalate to femicide, and intimate partner sexual violence and coercive control are recognised markers of escalation risk. Shortly after fieldwork concluded, Chile enacted Law 21.212 (“Ley Gabriela”, 2020), which broadened the legal definition of femicide to include killings by current or former partners without cohabitation and certain non-partner gender-based killings.^7^

An estimated 840 million women and adolescent girls worldwide have experienced physical violence, sexual violence, or both by an intimate partner or sexual violence by a non-partner at least once in their lifetime, with little progress in the past two decades.^8^ Globally, 24.7% of ever-partnered women aged 15 years and older have experienced physical or sexual intimate partner violence during their lifetime, and 8.2% have experienced non-partner sexual violence.^9^ The WHO 2025 report calls for more comprehensive and standardised measures, including disaggregation by contact versus non-contact forms and by perpetrator type, since most surveys feeding global estimates have historically measured only rape and attempted rape.^9^ Population-based studies in high-income settings using broader operational definitions report substantially higher prevalences: in Spain, lifetime prevalence among women aged 16 years and older was 9.2% for partner sexual violence, 6.5% for non-partner sexual assault, and 40.4% for sexual harassment.^5^ These figures are likely underestimates given barriers to disclosure,^10^ and the health consequences are substantial, including post-traumatic stress disorder,^11^ depression and anxiety symptoms following both contact and non-contact forms,^12^ with intimate partner violence alone accounting for a large share of the global mental health burden among women,^13^ and significant costs to health systems.^14^

We adopted Heise's ecological framework,^15^^,^^16^ which identifies four levels of influence (individual, relationship, community, and societal) later taken up by WHO.^2^ Cross-national evidence consistently links higher victimisation to lower women's status and greater gender inequality^17^; multi-country analyses identify male dominance as a key correlate,^18^ and national studies point to younger age, being unpartnered, and exposure to other forms of violence.^19^ Multilevel analyses, however, have focused almost exclusively on intimate partner violence, mostly in sub-Saharan Africa,^18^^,^^20^^,^^21^ and rarely distinguish forced physical contact from non-contact harassment. In Chile, a recent World Bank report identified young age, low education, disability, childhood sexual abuse, and partner aggression as the main risk factors for intimate partner violence,^22^ but the multilevel determinants of sexual violence in Latin America remain underexamined, and far less is known about non-partner forms. A recent editorial in this journal underscored that little is known about trends in the prevalence of physical or sexual violence in the Americas, calling for stronger regional evidence.^23^

We aimed to characterise the social determinants of three forms of sexual violence, IPSV, NPSA, and NPSH, in Chile, and to explore whether their risk profiles differ across forms.

## Methods

### Study design and data source

We did a cross-sectional, secondary analysis of the Chilean National Survey on Violence in the Family and Other Settings (ENVIF), administered by the Subsecretariat for Crime Prevention. We pooled the 2017 and 2020 rounds, as municipal identifiers are not available in the publicly released 2024 dataset. The ENVIF asks about specific behaviours rather than general experiences and includes a self-administered section to enhance privacy and reduce underreporting,^10^ following WHO recommendations.^9^ The study followed the Strengthening the Reporting of Observational Studies in Epidemiology (STROBE) statement.

### Population and sampling

The target population was women residing in urban areas of Chile, aged 15–65 years in the 2017 round and 15 years or older in the 2020 round; the 2020 round therefore includes 960 women aged 66 years or older with no counterpart in 2017. Urban areas, as classified by the National Institute of Statistics (INE), held 87.8% of the Chilean population at the 2017 Census, so the survey covers the large majority of Chilean women but not those in rural settings. Both samples are probabilistic, drawn using a three-stage stratified cluster design (blocks; households; and individual respondent) stratified by region, so that estimates are representative of the urban female population nationally and within each region, which is what “regionally representative” denotes. The two rounds used different dwelling frames from INE: the 2017 round was drawn from the 2002 census-based housing frame with continuously updated cartography, as the 2017 census frame was not yet available for survey design at the time, whereas the 2020 round was drawn from the 2017 Master Sampling Frame of Dwellings (MMV 2017), built from the definitive 2017 Population and Housing Census. ENVIF 2017 sampled 230 municipalities and ENVIF 2020 a more concentrated set of 149; the pooled sample spans 244 municipalities. The 2017 wave was administered between December 6, 2016 and March 9, 2017; the 2020 wave between December 1, 2019 and March 2, 2020, with fieldwork concluding before the first confirmed COVID-19 case in Chile (March 3, 2020). In line with the Sex and Gender Equity in Research (SAGER) guidelines, we describe how sex and gender were ascertained. ENVIF is a survey of women: respondents were selected within each household by the interviewer from among resident women aged 15 years or older. The questionnaire contains no item on the respondent's sex assigned at birth or gender identity. “Women” therefore denotes the interviewer-ascertained target population rather than a measured variable, and cisgender and transgender women cannot be distinguished.

### Outcomes

We constructed three dichotomous outcomes capturing victimisation in the previous 12 months, distinguishing perpetrator type and the presence of forced physical contact (Supplementary Table S1).^5^ We chose a past-year window rather than lifetime victimisation because the analysis of social determinants requires temporal alignment between exposures and outcome: lifetime measures conflate determinants that may have changed since the event, including municipality of residence, age and partnership status at the time of victimisation. A past-year window also reduces recall bias and improves cross-wave comparability.

IPSV was defined using the five items administered identically or near-identically in both waves (forced intercourse, ‘forced you to have sexual intercourse when you did not want to’; physically forced intercourse, ‘physically forced you to have sexual intercourse when you did not want to’; humiliating or degrading sexual act; threat to withhold household money conditioned on sex; forced discontinuation of contraception), so that the outcome is measurement-invariant across the pooled sample. The 2020 instrument added three items (forced viewing of pornography, forced unprotected sex, and forced recording of sexual content); a sensitivity analysis using the full eight-item 2020 battery is reported in the Supplementary Material. The denominator was restricted to women with a partner in the previous 12 months. The two rounds differed in the administration of this battery: in 2017 it was asked only of women with a current partner, whereas in 2020 it was asked of all respondents. Women whose only partner in the previous 12 months was a former partner were therefore not asked in 2017 (288 women, 5.9% of the 2017 IPSV denominator); they are treated as missing rather than as non-victims, and IPSV prevalence is not strictly comparable between rounds. NPSA was derived from eight items capturing forced physical sexual contact by non-partners across public spaces, workplaces, and educational settings, corresponding to the strict WHO definition of non-partner sexual violence.^3^^,^^9^ NPSH was derived from six items capturing non-contact behaviours (obscene verbal advances, intimidation, and quid-pro-quo propositions or retaliation) across the same settings.^3^^,^^5^ Items are listed in Supplementary Table S1.

For each item, the 12-month reference period was operationalised through a dedicated filter question that followed the lifetime screening item and asked whether the event had occurred in the past 12 months (intimate partner sexual violence: 2017 P73.B, 2020 P132.B; public spaces: 2017 P94, 2020 P72; workplace: 2017 P102, 2020 P82; and educational settings: 2017 P87, 2020 P94). A woman was classified as a victim of a given form of violence if she answered ‘yes’ to that 12-month filter for at least one component item. The NPSA and NPSH denominators included the full sample; women could be positive for any combination of the three outcomes.

### Independent variables

Covariates were organised following the ecological framework (Supplementary Table S2). Individual-level covariates were age, education, socioeconomic status, marital status, social participation, Indigenous identity, disability, own income, childhood sexual abuse, and an Individual Gender Index. The Individual Gender Index comprised 14 items in 2017 (Cronbach alpha 0.90) and 13 items in 2020 (alpha 0.75); the item “If a couple cannot agree on something, the final decision should be made by the man if he is the one who provides for the household” was dropped in 2020 (Supplementary Table S3). Higher scores mean more traditional attitudes. The index was z-standardised within survey wave, so its coefficient reflects a woman's relative position within her own wave rather than an absolute score. Relationship-level covariates (IPSV models only) were dominant relationship dynamics (Supplementary Table S4), partner exposure to violence in childhood and partner violence towards others. For the partner's exposure to violence in childhood, responses of ‘don't know’ were coded as no known exposure and ‘no answer’ as missing, identically in both rounds. Community-level covariates were municipal poverty rate (small-area income-poverty estimates from the National Socioeconomic Characterisation Survey, CASEN: 2017 estimates for the 2017 wave and 2020 estimates, 2022 revision, for the 2020 wave), the municipal rate of crimes of greater social connotation (delitos de mayor connotación social) per 100,000 inhabitants (Centre of Studies and Crime Analysis, CEAD: 2017 and 2019 rates respectively, under an identical definition in both waves) and the average municipal gender index, computed within each wave. At the societal level, we included residence in the Metropolitan Region (Santiago) versus other regions. Models M2 and M3 adjusted for survey wave.

Partner alcohol consumption (29.3% missing among partnered women; 56.2% in 2020), partner education (38.4%; 68.5% in 2020) and partner age difference (6.7%) were excluded a priori due to severe deterioration in item administration in the 2020 round (Supplementary Table S5); migration status was available only in 2020. Age was categorised into seven groups (15–18 [reference], 19–25, 26–35, 36–45, 46–55, 56–65, and ≥66; the last group comprises 2020 respondents only). The Individual Gender Index was standardised to z-scores within year, and the municipal covariates on the pooled analytic sample. The Pearson correlation matrix of the covariates (Supplementary Table S6) is retained as descriptive, because most covariates are binary or categorical and product–moment correlations do not index linear dependence for them; multicollinearity was assessed primarily through variance inflation factors from the fitted models (Supplementary Table S7).

### Ethics

The protocol was approved by the Comité de Ética de Investigación en Seres Humanos, Universidad de Chile (N° 014-2025); the analysis used the publicly available, fully anonymised ENVIF dataset.

### Statistical analysis

We fitted multilevel mixed-effects logistic regression separately for each outcome, with women (level 1) nested within municipalities (level 2). The multilevel specification addresses three substantive concerns: clustering within municipalities, which would otherwise underestimate standard errors of contextual covariates; the nested structure of the ecological framework; and the variance partition coefficient as a substantive parameter quantifying between-municipality heterogeneity. Partner-level variables are meaningful only for IPSV, justifying structurally different covariate sets across outcomes. The primary sampling unit (block) could not serve as level 2 (only four to five women per block).^24^ For each outcome we fitted four nested models: M0 (empty), M1 (individual), M2 (contextual), and M3 (full).

Prevalence estimates are weighted using the ENVIF post-stratification weights supplied with each round, which calibrate the sample to official population projections; numerators report unweighted case counts. Odds ratios (ORs) with 95% confidence intervals (CIs) were estimated and significance assessed using the Wald test (α = 0.05 two-sided). Between-municipality heterogeneity was summarised through the variance partition coefficient. Goodness of fit was assessed using the Akaike information criterion and the Bayesian information criterion. Models were fitted by maximum likelihood with the Laplace approximation (one quadrature point); for the low-prevalence IPSV outcome, robustness to the integration method was confirmed by re-estimating the fully adjusted model with adaptive Gauss–Hermite quadrature (ten quadrature points; Supplementary Table S8). Confidence intervals for the variance partition coefficient were obtained by profile likelihood (Supplementary Table S8). Missing data were handled by complete-case analysis: missingness was below 5% for all retained covariates (Supplementary Table S9) except dominant relationship (6.0%; Supplementary Table S5). We compared women included in and excluded from the IPSV analytic sample (Supplementary Table S10) and re-estimated the fully adjusted IPSV model under multiple imputation by chained equations (m = 20 imputations; Supplementary Table S11). Bivariate associations (Supplementary Tables S12–S14) are reported on the maximal available sample for each outcome to preserve power and minimise selection bias in unadjusted comparisons; multivariable models use the analytic complete-case samples described above. Primary models were unweighted. Survey weights were used to estimate population prevalences; the models target conditional associations, for which weighting is not required under a correctly specified model and can substantially inflate variance components in multilevel specifications.

As sensitivity analyses, all models were re-estimated with two complementary specifications: survey-weighted multilevel models using rescaled ENVIF post-stratification weights (WeMix package; Supplementary Tables S15–S17), preserving representativeness under potentially informative missingness; and single-level logistic regressions (Supplementary Tables S18–S20), to verify that conclusions do not hinge on the multilevel specification. To test the homogeneity of associations across waves, we additionally estimated all models separately within each wave (Supplementary Table S21) and compared the fully adjusted models with and without survey-wave interactions for the strongest determinants (dominant relationship, childhood sexual abuse, Metropolitan Region, and age) using likelihood-ratio tests (Supplementary Table S22). Analyses were done in R version 4.4.3 using lme4 (1.1-37), WeMix (4.0.3), survey (4.4-2), mice (3.19.0), and logistf (1.26.1); the full analytic code is publicly available on Figshare (https://doi.org/10.6084/m9.figshare.33273933).

### Role of the funding source

The funder had no role in study design, analysis, interpretation, writing, or the decision to submit.

## Results

### Sample characteristics

The combined ENVIF 2017 and 2020 rounds yielded 14,559 women aged 15 years or older across 244 municipalities (mean approximately 60 women per municipality; range 4–842). Three nested denominators were used in the analysis: the full pooled sample (n = 14,559; 244 municipalities) for unadjusted bivariate comparisons (Supplementary Tables S12–S14; for IPSV, the 9681 partnered women who were administered the battery); the NPSA and NPSH analytic sample (n = 13,128; 243 municipalities), after exclusion of women with missing individual-level covariates; and the IPSV analytic sample (n = 8335; 241 municipalities), restricted to the 9969 women (68.5% of the pooled sample) who reported a partner in the previous 12 months and further reduced after exclusion for missing partner-level covariates (Table 1). The participant flow is shown in Supplementary Figure S1. Mean age was 41.3 years (standard deviation [SD] 16.8). Percentages below are survey-weighted and counts are unweighted numbers of women: 48.0% had low socioeconomic status (7305 of 14,559), 41.5% had completed secondary education (5953 of 14,537), and 45.2% lived in the Metropolitan Region (2240 of 14,559). Indigenous identity was reported by 9.2% (1923 of 14,215), a long-term disability by 8.2% (1386 of 14,339), and sexual abuse before age 14 by 11.9% (1570 of 14,417). Among women with a partner, 18.3% (1460 of 9374) described their relationship as dominant. Mean municipal income-poverty was 9.3% (SD 4.1) and the mean rate of crimes of greater social connotation was 3180 per 100,000 inhabitants (SD 1336). No pairwise Pearson correlation between covariates exceeded 0.50 (Supplementary Table S6).

**Table 1:** Characteristics of the pooled sample.

Variables	Mean (SD) or % (unweighted n)	Range
Individual and household level		
Age (years)	41.3 (16.8)	15–95
Age group		
15–18	7.2 (543)	
19–25	14.3 (1501)	
26–35	20.7 (2629)	
36–45	18.2 (2611)	
46–55	18.0 (2927)	
56–65	14.0 (3388)	
66 or more	7.7 (960)	
Socioeconomic level		
Low	48.0 (7305)	
Medium	43.9 (6266)	
High	8.0 (988)	
Education		
None	9.8 (1681)	
Primary	24.0 (3639)	
Secondary	41.5 (5953)	
Tertiary	24.8 (3264)	
Married or in a civil union	35.1 (5685)	
Metropolitan Region	45.2 (2240)	
Indigenous identity	9.2 (1923)	
Disability	8.2 (1386)	
Childhood sexual abuse	11.9 (1570)	
Individual Gender Index	26.4 (7.8)	13–70
Own income	58.8 (8595)	
Social participation	38.5 (6277)	
Relationship level		
Dominant relationship	18.3 (1460)	
Partner: violence in childhood	26.1 (2485)	
Partner: violent in other settings	4.3 (389)	
Municipal level		
Municipal Gender Index	27.0 (3.7)	14–45
Municipal poverty rate	9.3 (4.1)	0.1–35.1
Municipal crime rate	3180 (1336)	456–9920

SD = standard deviation; CASEN = Encuesta de Caracterización Socioeconómica Nacional (National Socioeconomic Characterisation Survey); ENVIF = Encuesta Nacional de Violencia Intrafamiliar y en Otros Espacios (National Survey on Violence in the Family and Other Settings).

ENVIF 2017 and 2020, weighted.

Weighted means (SD) and percentages, accounting for the complex survey design. Range refers to the observed minimum and maximum for continuous variables; binary and categorical variables are coded 0/1 and summarised as percentages. Unweighted numbers of women are given in parentheses after each percentage; their denominators exclude missing values (Supplementary Tables S5 and S9). Relationship-level variables refer to women who reported a partner in the last 12 months; all other variables refer to the full pooled sample. Municipal poverty from the CASEN survey (Ministry of Social Development); municipal crime rate from the Centro de Estudios y Análisis del Delito; the Municipal Gender Index is the mean of the Individual Gender Index across women in each municipality. ENVIF is not a survey of race; it records self-identified Indigenous belonging (nine peoples legally recognised at the time of the surveys, plus other self-identified Indigenous belonging).

### Prevalence of the three forms

Past-year prevalence was 2.8% (95% CI 2.4–3.3) for IPSV, 2.5% (2.1–3.0) for NPSA, and 11.3% (10.4–12.3) for NPSH (Table 2). NPSH was nearly five-fold more prevalent than NPSA and four-fold more prevalent than IPSV, while NPSA and IPSV were of comparable magnitude. NPSH nearly doubled between waves (7.5%–14.7%) and NPSA rose more modestly (2.0%–2.9%). IPSV was 2.1% in 2017 and 3.5% in 2020, but because the battery was administered to a narrower population in 2017 (Methods), this difference should not be read as a trend. Because the 2020 wave was administered before the first confirmed COVID-19 case in Chile, the NPSA and NPSH increases pre-date the pandemic. Supplementary Figure S2 shows prevalences by age group and region.

**Table 2:** Past-12-month prevalence of the three outcomes.

Outcome	2017, % (95% CI)	2020, % (95% CI)	Pooled 2017–2020, % (95% CI)
Intimate partner sexual violence (IPSV)	2.1 (1.5–2.8) 82/4568	3.5 (2.8–4.2) 189/5113	2.8 (2.4–3.3) 271/9681
Non-partner sexual assault (NPSA)	2.0 (1.4–2.7) 59/6824	2.9 (2.3–3.5) 153/7735	2.5 (2.1–3.0) 212/14,559
Non-partner sexual harassment (NPSH)	7.5 (6.2–8.9) 281/6824	14.7 (13.3–16.0) 823/7735	11.3 (10.4–12.3) 1104/14,559

CI = confidence interval; IPSV = intimate partner sexual violence; NPSA = non-partner sexual assault; NPSH = non-partner sexual harassment; ENVIF = Encuesta Nacional de Violencia Intrafamiliar y en Otros Espacios (National Survey on Violence in the Family and Other Settings).

ENVIF 2017 and 2020, weighted.

Weighted prevalences with 95% confidence intervals, estimated using survey weights and clustering at the primary sampling unit. IPSV denominator: women who reported a partner in the last 12 months. NPSA and NPSH denominator: full sample. IPSV is defined using the five items asked identically or near-identically in both waves. Below each estimate, the unweighted number of women reporting the outcome over the unweighted denominator (cases/n). The IPSV denominator excludes 288 women in 2017 for whom the battery was not administered (Methods).

### Multilevel models for intimate partner sexual violence

Across the four IPSV models (Table 3; n = 8335 women in 241 municipalities), the municipal-level variance partition coefficient was effectively zero (M0–M3: 0.00%; profile-likelihood 95% CI at M3 0.00–2.57; Supplementary Table S8b), indicating no detectable between-municipality clustering. Adding individual covariates substantially improved fit (Akaike information criterion: M0 = 1884; M1 = 1625), whereas the contextual-only model showed almost no improvement (M2 = 1877), and the full model closely tracked M1 (M3 = 1625). Sensitivity analyses yielded substantively equivalent estimates (Supplementary Tables S8, S15, S18, S23 and S24). The fully adjusted model used 199 outcome events across 26 parameters (events per variable 7.7); Firth penalised estimates were materially unchanged (Supplementary Table S24c).

**Table 3:** Multilevel logistic regression for intimate partner sexual violence.

Variables	M0	M1	M2	M3
Intercept				
Intercept (baseline odds)	0.02 (0.02–0.03) ∗∗∗	0.02 (0.01–0.04) ∗∗∗	0.02 (0.01–0.02) ∗∗∗	0.02 (0.01–0.04) ∗∗∗
Level 1 - Individual				
Age group (ref: 15–18)				
19–25		0.84 (0.36–1.96)		0.84 (0.36–1.97)
26–35		0.46 (0.20–1.07)		0.46 (0.20–1.07)
36–45		0.51 (0.22–1.20)		0.51 (0.22–1.20)
46–55		0.46 (0.20–1.09)		0.45 (0.19–1.06)
56–65		0.49 (0.21–1.17)		0.47 (0.20–1.13)
66 or more		0.10 (0.02–0.53) ∗∗		0.09 (0.02–0.46) ∗∗
Socioeconomic level (ref: Low)				
Medium		0.84 (0.61–1.16)		0.90 (0.65–1.26)
High		1.30 (0.65–2.57)		1.35 (0.67–2.69)
Education (ref: None)				
Primary		1.21 (0.71–2.09)		1.15 (0.67–1.99)
Secondary		1.02 (0.59–1.78)		0.94 (0.54–1.64)
Tertiary		0.61 (0.31–1.22)		0.56 (0.28–1.11)
Married or in a civil union		1.18 (0.83–1.67)		1.21 (0.85–1.72)
Indigenous identity		1.12 (0.75–1.68)		1.08 (0.71–1.62)
Disability		1.44 (0.89–2.32)		1.37 (0.85–2.23)
Childhood sexual abuse		2.09 (1.49–2.95) ∗∗∗		2.05 (1.45–2.89) ∗∗∗
Individual Gender Index (z)		1.03 (0.88–1.20)		1.04 (0.88–1.24)
Own income		0.84 (0.62–1.14)		0.84 (0.61–1.14)
Social participation		1.13 (0.83–1.52)		1.07 (0.79–1.45)
Level 1 - Relationship				
Dominant relationship		6.36 (4.66–8.69) ∗∗∗		6.40 (4.68–8.76) ∗∗∗
Partner: violence in childhood		1.58 (1.16–2.16) ∗∗		1.60 (1.17–2.18) ∗∗
Partner: violent in other settings		2.85 (1.80–4.52) ∗∗∗		2.79 (1.75–4.43) ∗∗∗
Level 2 - Municipality				
Metropolitan Region			0.78 (0.50–1.22)	0.61 (0.38–0.96) ∗
Municipal Gender Index (z)			1.05 (0.89–1.24)	1.00 (0.83–1.21)
Municipal poverty rate (z)			1.08 (0.92–1.27)	0.98 (0.82–1.16)
Municipal crime rate (z)			1.00 (0.86–1.17)	1.02 (0.86–1.21)
Level 2 - Survey wave				
Survey wave: 2020 (ref: 2017)			1.60 (1.18–2.16) ∗∗	1.41 (1.02–1.95) ∗
Model fit				
n (women)	8335	8335	8335	8335
n (municipalities)	241	241	241	241
AIC	1884	1625	1877	1625
BIC	1898	1787	1926	1822
Log-likelihood	−939.9	−789.6	−931.4	−784.5
VPC, municipality (%)	0.00	0.00	0.00	0.00
Median odds ratio	1.00	1.00	1.00	1.00

M0 = empty model (municipality random intercept only); M1 = M0 plus individual-level and relationship-level covariates; M2 = M0 plus contextual covariates (Metropolitan Region and municipal-level variables) and survey wave; M3 = full model with all covariates. OR = odds ratio; CI = confidence interval; ref = reference category; (z) = z-standardised variable (Individual Gender Index within survey wave; municipal covariates on the pooled analytic sample); AIC = Akaike information criterion; BIC = Bayesian information criterion; VPC = variance partition coefficient; ENVIF = Encuesta Nacional de Violencia Intrafamiliar y en Otros Espacios (National Survey on Violence in the Family and Other Settings). Confidence limits are shown to two decimal places; a limit displayed as 1.00 may still exclude the null value, as indicated by the significance marker.

ENVIF 2017 and 2020, women with a partner in the last 12 months.

OR (95% CI). Significance: ∗∗∗p < 0.001; ∗∗p < 0.01; ∗p < 0.05.

Relationship-level characteristics emerged as the strongest correlates of IPSV (M3). Women describing their relationship as dominant had the largest adjusted association with IPSV (OR 6.40, 95% CI 4.68–8.76). Having a partner violent towards others (2.79, 1.75–4.43) and a partner with childhood exposure to violence (1.60, 1.17–2.18) were also strongly associated. A history of childhood sexual abuse doubled the odds (2.05, 1.45–2.89). Disability showed a positive but imprecise association that did not reach conventional significance (1.37, 0.85–2.23). Marital status, own income, Indigenous identification, education, socioeconomic level, social participation, and the Individual Gender Index showed weak or null associations after adjustment. IPSV odds were higher in 2020 than in 2017 (1.41, 1.02–1.95); the Metropolitan Region indicator was borderline (0.61, 0.38–0.96), and no municipal contextual variable was associated with IPSV (poverty 0.98, 0.82–1.16; crime 1.02, 0.86–1.21).

Results were largely robust to the outcome definition: under the eight-item 2020 battery, 2020 prevalence was 4.1% (versus 3.5% with the harmonised definition) and the fully adjusted survey-wave association was OR 1.53 (1.11–2.10) versus 1.41 (1.02–1.95). The only coefficient whose interpretation changed was age 26–35 years, which reached significance under the eight-item definition (0.43, 0.19–0.97) but not under the harmonised one (0.46, 0.20–1.07); disability lay just above one under both definitions (Supplementary Table S25). Compared with included women, excluded women were younger and less educated (Supplementary Table S10). Under multiple imputation, estimates were consistent with the complete-case analysis for the individual-level determinants and for dominant relationship (6.30, 4.71–8.43), while the association with having a partner violent towards others strengthened, to 4.06 (2.82–5.85), and the survey-wave association was 1.52 (1.14–2.03) (Supplementary Table S11).

### Multilevel models for non-partner sexual assault

Across the four NPSA models (Table 4; n = 13,128), the empty-model variance partition coefficient was 8.31%, indicating moderate between-municipality clustering. M1 left it essentially unchanged (8.40%); M2 reduced it to 3.55% and M3 to 3.33% (95% CI 0.00–9.52), indicating that contextual rather than individual variables accounted for most of the initial clustering. Sensitivity analyses yielded broadly similar estimates (Supplementary Tables S16 and S19).

**Table 4:** Multilevel logistic regression for non-partner sexual assault.

Variables	M0	M1	M2	M3
Intercept				
Intercept (baseline odds)	0.01 (0.01–0.02) ∗∗∗	0.03 (0.01–0.06) ∗∗∗	0.01 (0.01–0.01) ∗∗∗	0.01 (0.01–0.03) ∗∗∗
Level 1 - Individual				
Age group (ref: 15–18)				
19–25		0.78 (0.44–1.38)		0.86 (0.49–1.51)
26–35		0.29 (0.15–0.53) ∗∗∗		0.32 (0.17–0.60) ∗∗∗
36–45		0.26 (0.14–0.50) ∗∗∗		0.30 (0.16–0.57) ∗∗∗
46–55		0.23 (0.12–0.44) ∗∗∗		0.25 (0.13–0.48) ∗∗∗
56–65		0.20 (0.10–0.38) ∗∗∗		0.21 (0.11–0.40) ∗∗∗
66 or more		0.06 (0.01–0.27) ∗∗∗		0.04 (0.01–0.19) ∗∗∗
Socioeconomic level (ref: Low)				
Medium		1.12 (0.81–1.53)		1.16 (0.84–1.60)
High		1.45 (0.81–2.61)		1.32 (0.73–2.38)
Education (ref: None)				
Primary		0.89 (0.45–1.78)		0.83 (0.42–1.65)
Secondary		1.17 (0.61–2.24)		1.02 (0.53–1.97)
Tertiary		0.86 (0.41–1.77)		0.68 (0.33–1.42)
Married or in a civil union		0.46 (0.30–0.69) ∗∗∗		0.47 (0.31–0.71) ∗∗∗
Indigenous identity		1.20 (0.82–1.77)		1.22 (0.84–1.79)
Disability		1.36 (0.79–2.32)		1.27 (0.74–2.16)
Childhood sexual abuse		3.44 (2.49–4.76) ∗∗∗		3.03 (2.20–4.20) ∗∗∗
Individual Gender Index (z)		0.86 (0.73–1.01)		0.85 (0.71–1.02)
Own income		1.18 (0.85–1.63)		1.15 (0.83–1.60)
Social participation		1.45 (1.09–1.95) ∗		1.49 (1.11–2.00) ∗∗
Level 2 - Municipality				
Metropolitan Region			2.60 (1.79–3.77) ∗∗∗	2.92 (2.00–4.28) ∗∗∗
Municipal Gender Index (z)			1.02 (0.85–1.22)	1.09 (0.90–1.33)
Municipal poverty rate (z)			1.06 (0.87–1.29)	1.03 (0.84–1.27)
Municipal crime rate (z)			1.11 (0.95–1.29)	1.10 (0.94–1.29)
Level 2 - Survey wave				
Survey wave: 2020 (ref: 2017)			2.21 (1.59–3.08) ∗∗∗	2.14 (1.51–3.03) ∗∗∗
Model fit				
n (women)	13,128	13,128	13,128	13,128
n (municipalities)	243	243	243	243
AIC	2063	1909	2017	1862
BIC	2078	2058	2069	2049
Log-likelihood	−1029.5	−934.3	−1001.4	−906.2
VPC, municipality (%)	8.31	8.40	3.55	3.33
Median odds ratio	1.68	1.69	1.39	1.38

M0 = empty model (municipality random intercept only); M1 = M0 plus individual-level covariates; M2 = M0 plus contextual covariates (Metropolitan Region and municipal-level variables) and survey wave; M3 = full model with all covariates. OR = odds ratio; CI = confidence interval; ref = reference category; (z) = z-standardised variable (Individual Gender Index within survey wave; municipal covariates on the pooled analytic sample); AIC = Akaike information criterion; BIC = Bayesian information criterion; VPC = variance partition coefficient; ENVIF = Encuesta Nacional de Violencia Intrafamiliar y en Otros Espacios (National Survey on Violence in the Family and Other Settings). Confidence limits are shown to two decimal places; a limit displayed as 1.00 may still exclude the null value, as indicated by the significance marker.

ENVIF 2017 and 2020, full pooled sample.

OR (95% CI). Significance: ∗∗∗p < 0.001; ∗∗p < 0.01; ∗p < 0.05.

A history of childhood sexual abuse was the strongest individual-level correlate (3.03, 2.20–4.20). A steep age gradient was observed, with odds declining monotonically from women aged 26–35 years (0.32, 0.17–0.60) to those ≥66 years (0.04, 0.01–0.19). Women who were married or in a civil union had lower odds (0.47, 0.31–0.71). Women who took part in social organisations had higher odds of NPSA (1.49, 1.11–2.00). Women in the Metropolitan Region had markedly higher odds (2.92, 2.00–4.28), and odds were more than twice as high in 2020 as in 2017 (2.14, 1.51–3.03).

### Multilevel models for non-partner sexual harassment

Across the four NPSH models (Table 5; n = 13,128), the empty-model variance partition coefficient was 8.27%; M1 left it essentially unchanged (8.22%), M2 reduced it to 4.16% and M3 to 4.34% (95% CI 2.17–7.62), the same pattern as NPSA, with contextual rather than individual variables accounting for the initial clustering. Sensitivity analyses yielded equivalent estimates (Supplementary Tables S17 and S20).

**Table 5:** Multilevel logistic regression for non-partner sexual harassment.

Variables	M0	M1	M2	M3
Intercept				
Intercept (baseline odds)	0.07 (0.06–0.08) ∗∗∗	0.25 (0.16–0.38) ∗∗∗	0.03 (0.03–0.04) ∗∗∗	0.13 (0.08–0.20) ∗∗∗
Level 1 - Individual				
Age group (ref: 15–18)				
19–25		0.56 (0.42–0.74) ∗∗∗		0.61 (0.45–0.81) ∗∗∗
26–35		0.23 (0.17–0.31) ∗∗∗		0.25 (0.19–0.34) ∗∗∗
36–45		0.13 (0.10–0.18) ∗∗∗		0.14 (0.10–0.20) ∗∗∗
46–55		0.11 (0.08–0.16) ∗∗∗		0.12 (0.08–0.16) ∗∗∗
56–65		0.06 (0.04–0.09) ∗∗∗		0.06 (0.04–0.09) ∗∗∗
66 or more		0.04 (0.02–0.08) ∗∗∗		0.03 (0.01–0.05) ∗∗∗
Socioeconomic level (ref: Low)				
Medium		0.83 (0.71–0.96) ∗		0.92 (0.78–1.07)
High		1.11 (0.85–1.45)		1.07 (0.81–1.41)
Education (ref: None)				
Primary		0.77 (0.53–1.11)		0.70 (0.48–1.01)
Secondary		1.16 (0.82–1.64)		1.01 (0.71–1.43)
Tertiary		1.46 (1.01–2.11) ∗		1.17 (0.80–1.70)
Married or in a civil union		0.71 (0.59–0.85) ∗∗∗		0.74 (0.62–0.89) ∗∗
Indigenous identity		1.12 (0.93–1.37)		1.11 (0.91–1.35)
Disability		1.36 (1.02–1.83) ∗		1.23 (0.92–1.66)
Childhood sexual abuse		2.34 (1.96–2.79) ∗∗∗		2.06 (1.72–2.46) ∗∗∗
Individual Gender Index (z)		0.68 (0.63–0.74) ∗∗∗		0.68 (0.63–0.75) ∗∗∗
Own income		1.22 (1.04–1.43) ∗		1.18 (1.00–1.38) ∗
Social participation		1.33 (1.15–1.53) ∗∗∗		1.26 (1.09–1.46) ∗∗
Level 2 - Municipality				
Metropolitan Region			1.97 (1.55–2.52) ∗∗∗	2.29 (1.77–2.96) ∗∗∗
Municipal Gender Index (z)			0.91 (0.83–1.00)	1.08 (0.98–1.20)
Municipal poverty rate (z)			1.15 (1.03–1.28) ∗	1.15 (1.02–1.29) ∗
Municipal crime rate (z)			1.15 (1.05–1.27) ∗∗	1.14 (1.03–1.27) ∗∗
Level 2 - Survey wave				
Survey wave: 2020 (ref: 2017)			2.71 (2.31–3.18) ∗∗∗	2.66 (2.24–3.16) ∗∗∗
Model fit				
n (women)	13,128	13,128	13,128	13,128
n (municipalities)	243	243	243	243
AIC	7125	6141	6891	5909
BIC	7140	6291	6943	6096
Log-likelihood	−3560.5	−3050.5	−3438.5	−2929.7
VPC, municipality (%)	8.27	8.22	4.16	4.34
Median odds ratio	1.68	1.68	1.43	1.45

M0 = empty model (municipality random intercept only); M1 = M0 plus individual-level covariates; M2 = M0 plus contextual covariates (Metropolitan Region and municipal-level variables) and survey wave; M3 = full model with all covariates. OR = odds ratio; CI = confidence interval; ref = reference category; (z) = z-standardised variable (Individual Gender Index within survey wave; municipal covariates on the pooled analytic sample); AIC = Akaike information criterion; BIC = Bayesian information criterion; VPC = variance partition coefficient; ENVIF = Encuesta Nacional de Violencia Intrafamiliar y en Otros Espacios (National Survey on Violence in the Family and Other Settings). Confidence limits are shown to two decimal places; a limit displayed as 1.00 may still exclude the null value, as indicated by the significance marker.

ENVIF 2017 and 2020, full pooled sample.

OR (95% CI). Significance: ∗∗∗p < 0.001; ∗∗p < 0.01; ∗p < 0.05.

The risk profile for NPSH parallelled NPSA in several dimensions but diverged in others. The age gradient was even steeper, with odds declining from women aged 26–35 years (0.25, 0.19–0.34) to those ≥66 years (0.03, 0.01–0.05). Childhood sexual abuse was again strongly associated (2.06, 1.72–2.46). Social participation was associated with a moderate increase in odds (1.26, 1.09–1.46). In contrast to NPSA, the Individual Gender Index was inversely associated with NPSH (0.68 per 1-SD increase, 0.63–0.75). Own income was associated with higher odds (1.18, 1.00–1.38), and being married or in a civil union with lower odds (0.74, 0.62–0.89). Women in the Metropolitan Region had higher odds (2.29, 1.77–2.96), and odds were higher in 2020 than in 2017 (2.66, 2.24–3.16). Survey-wave interactions did not improve fit for IPSV (likelihood-ratio test chi-square = 6.60, 8 degrees of freedom, p = 0.58); for NPSA and NPSH, interaction models fitted better (p = 0.031 and p = 0.0086), driven by a Metropolitan Region association that was stronger in 2017 than in 2020, with the other tested determinants stable across waves (Supplementary Tables S21 and S22).

Fig. 1 displays the adjusted odds ratios from the M3 models side-by-side for the three outcomes, highlighting the qualitatively different risk profiles: IPSV is associated mainly with relational and life-course factors, with negligible between-municipality clustering and a single isolated contextual association (Metropolitan Region), whereas NPSA and NPSH share a steep age gradient and territorial concentration in the Metropolitan Region. Childhood sexual abuse emerges as a shared upstream determinant of all three forms.

**Fig. 1:**
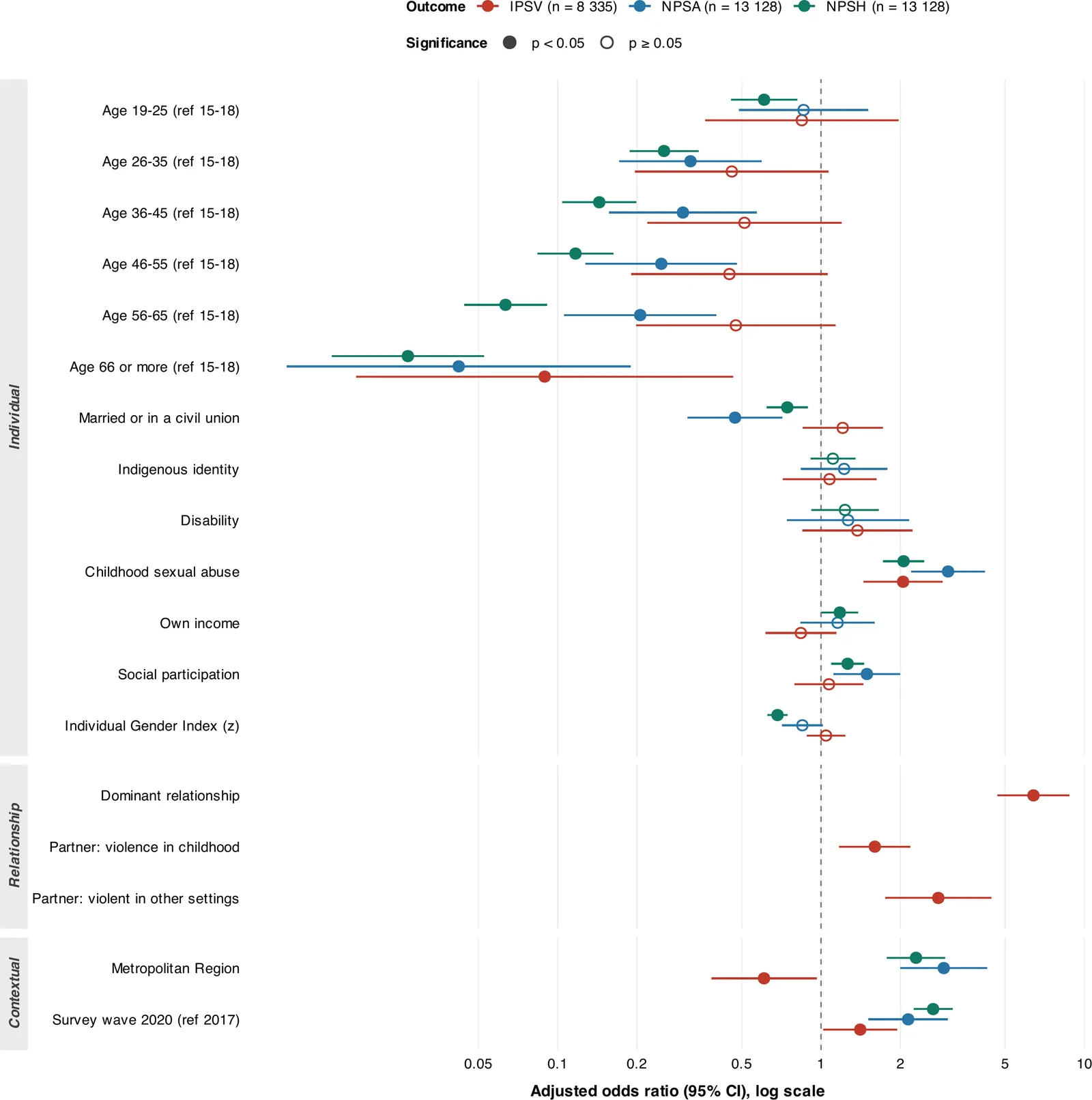
**Adjusted odds ratios for the three forms of sexual violence.** Adjusted odds ratios (OR) with 95% confidence intervals (CI) from the fully adjusted multilevel logistic models (M3, including individual, relationship and contextual covariates and survey wave) for intimate partner sexual violence (IPSV; n = 8335 women with a partner in the previous 12 months), non-partner sexual assault (NPSA; n = 13,128) and non-partner sexual harassment (NPSH; n = 13,128). Data from the pooled 2017 and 2020 rounds of the Chilean National Survey on Violence in the Family and Other Settings (ENVIF). Reference categories: age 15–18 years; survey wave 2017. Relationship-level covariates apply to IPSV only. Socioeconomic level, education and the municipal-level covariates (poverty rate, crime rate and Municipal Gender Index) are included in all models but not displayed; complete estimates are given in Table 3, Table 4, Table 5. (z) denotes variables z-standardised within survey wave. Filled circles indicate p < 0.05; open circles, p ≥ 0.05. Log scale; dashed line at OR = 1.

## Discussion

We identified no previous population-based multilevel analysis in Latin America that contrasts IPSV, NPSA and NPSH within a single framework. The three forms show distinct social patterns: IPSV is dominated by relational dynamics, with no detectable between-municipality clustering; NPSA and NPSH share a steep age gradient and territorial concentration in the Metropolitan Region, yet diverge in their association with gender attitudes. The clear prevalence gradient (NPSH 11.3%; IPSV 2.8%; NPSA 2.5%) and the differential risk profiles support the WHO call for disaggregated, standardised measures of sexual violence,^9^ and indicate that aggregating these forms, as much of the Latin American literature still does, obscures their social distribution and the policy responses they require.

For IPSV, the factor most strongly associated was a dominant relationship (OR 6.40, 95% CI 4.68–8.76), followed by three relational and life-course factors: partner violence in other settings, partner exposure to violence in childhood, and the woman's own history of childhood sexual abuse. Because the analysis is cross-sectional, estimates describe associations rather than causal effects. This matters most for the dominant-relationship variable: coercive control and intimate partner sexual coercion are closely related manifestations of partner violence, so this strong association may partly reflect construct overlap or reverse temporality, and should be read as a marker of a controlling relationship dynamic rather than an independent cause. These findings are consistent with international evidence suggesting that partner sexual violence is associated more with relational dynamics than with structural socioeconomic indicators^18^^,^^25^ and converge with the only previous Chilean analysis of comparable scope.^22^ Childhood sexual abuse may be associated with a higher probability of partnering with men exhibiting dominant or violent behaviour, through partner selection, normalisation of coercive dynamics, and reduced threat recognition.^26^ Disability was not associated with any of the three outcomes after adjustment, although the IPSV point estimate lay above one with a wide interval (1.37, 0.85–2.23). Meta-analytic evidence reports substantially higher sexual victimisation among women with disabilities,^27^ so we read this as inconclusive rather than as evidence of no association: with 199 events, the model has limited power to detect a moderate effect for a characteristic present in 8.2% of the sample. The negligible between-municipality clustering likely reflects Chile's homogeneous national legal framework and a municipal unit too coarse to capture the smaller-scale contexts in which intimate relationships are shaped: IPSV appears largely non-territorial in this setting, distributed according to the relational composition of municipal populations rather than municipal-level determinants. Because the variance estimate lies at the boundary of the parameter space, we read this as an upper bound rather than a point estimate: the profile-likelihood interval and the survey-weighted specification both place any between-municipality component at a small magnitude (Supplementary Tables S8b and S15).

For NPSA and NPSH, risk concentrated among younger women and in the Metropolitan Region, replicating patterns documented elsewhere^28^ and consistent with the concentration of sexual victimisation in transit-intensive urban environments.^29^ The steep age gradients most plausibly reflect differential exposure: younger women spend more time in the public, occupational, and night-time spaces where non-partner perpetrators are encountered. The same logic extends to social participation, which was associated with higher odds of both non-partner outcomes (NPSA 1.49, 1.11–2.00; NPSH 1.26, 1.09–1.46) but not of IPSV. Taking part in organisations places women in public, community, and workplace settings where non-partner perpetrators are encountered; we read this as a marker of exposure, and the implication is safer public and organisational environments, not reduced participation. This exposure framework alone, however, does not account for two contrasts between NPSA and NPSH.

First, own income was associated with higher odds of NPSH (OR 1.18, 1.00–1.38) but not NPSA (1.15, 0.83–1.60). This contrast argues against a generic exposure account, since both forms would be expected to rise with time outside the domestic sphere. NPSH concentrates in workplaces and educational settings, where women in paid employment are over-represented and asymmetric power dynamics both increase exposure and improve recognition of non-consensual behaviours^28^; NPSA occurs more often in transit and anonymous public spaces, where the link with income is weaker. Second, municipal poverty and crime rates showed small positive associations with NPSH only (OR per 1-SD 1.15, 1.02–1.29, and 1.14, 1.03–1.27), with null associations for NPSA (1.03, 0.84–1.27, and 1.10, 0.94–1.29) and IPSV, consistent with non-contact harassment being the form most closely tied to the general territorial conditions in which women circulate daily, whereas IPSV unfolds in the domestic sphere. The Metropolitan Region concentration also attenuated between 2017 and 2020, so pooled estimates for NPSA and NPSH average a stronger 2017 and a weaker 2020 association (Supplementary Table S21).

The same recognition-and-exposure logic helps interpret the inverse association between the Individual Gender Index and NPSH, but not NPSA. Women holding more traditional gender attitudes reported lower odds of harassment, which most plausibly reflects differential recognition and labelling of non-consensual behaviours rather than higher true risk. The contrast between NPSH (inverse) and NPSA (null) is itself informative: acts involving forced physical contact are less subject to differential labelling than non-contact harassment.

In the adjusted models, the odds of all three outcomes were higher in 2020 than in 2017 (ORs 1.41–2.66). For IPSV this comparison is not affected by the difference in administration described in the Methods: the analytic sample requires the dominant-relationship items, which were asked only of women with a current partner in both rounds. Because the 2020 ENVIF was administered before the first confirmed COVID-19 case in Chile, this increase pre-dates pandemic dynamics. It coincided with unprecedented feminist mobilisation in Chile, including the 2018 mayo feminista^30^ and the diffusion of #NiUnaMenos, #MeToo, and Las Tesis. Three non-mutually-exclusive mechanisms are plausible: a real increase in incidence; growing willingness to disclose, as feminist mobilisation provided new vocabularies for naming non-consensual experiences; and methodological refinements in the 2020 instrument. The smaller increases for NPSA (1.5-fold; adjusted OR 2.14) relative to NPSH (2-fold; adjusted OR 2.66), and the modest adjusted survey-wave association for IPSV (OR 1.41), are consistent with the disclosure interpretation: acts involving forced physical contact have a more stable recognition threshold. Distinguishing these mechanisms will require analyses with the 2024 ENVIF wave.

Strengths include the population-based design; behaviour-specific items consistent with WHO recommendations and aligned with the WHO 2025 call for disaggregated measures^9^; and a multilevel framework relevant for policies designed at municipal or regional scales. Limitations are substantive. Measuring sexual violence is inherently challenging owing to stigma; misclassification likely biases some estimates towards the null, and the survey does not capture sexual violence by family members other than a partner. The partner items were administered with a different response format in the two rounds (a frequency scale in 2017, a yes/no item in 2020), which we addressed through a 2020-only sensitivity analysis (Supplementary Table S23); coding ‘don't know’ responses on the partner's childhood exposure to violence as no known exposure may bias that association towards the null. More fundamentally, pooling the 2017 and 2020 rounds combines instruments of differing reliability and administration within a rapidly changing social context, including the 2018–2019 feminist mobilisations; we regard this as a methodological limitation of the pooled design, addressed through wave-stratified models and harmonised-item sensitivity analyses, and not merely as an interpretive explanation for the observed increase. The update of the INE dwelling frame between rounds (from the 2002-census basis to the 2017-census basis) is a further between-wave design change; wave-specific post-stratification weights and adjustment for survey wave mitigate, but cannot fully remove, its effect on comparability. Restricting the IPSV denominator to women partnered in the past 12 months also conditions on current partnership status; if relationship dissolution is itself a consequence of prior sexual violence, this could induce collider (selection) bias of uncertain direction. Municipal poverty and crime showed small positive associations with NPSH only, and municipal gender norms showed no robust association with any outcome; these null findings should be interpreted cautiously, as gradients may be masked by differential underreporting or operate at finer scales than the municipality. For IPSV the municipality-level variance was estimated at its boundary (singular fit); the profile-likelihood interval (variance partition coefficient 0.00%, 95% CI 0.00–2.57 in the fully adjusted model) is compatible with at most a small between-municipality component, and the near-zero clustering should be interpreted with this uncertainty in mind (Supplementary Table S8). The IPSV instrument was expanded in 2020. We addressed this by defining the primary outcome on the five items common to both waves; the eight-item sensitivity analysis yielded consistent associations, although the harmonised definition necessarily understates the fuller spectrum of intimate partner sexual violence measured in 2020. Representativeness is bounded in two ways. The survey covers urban women only, so the findings do not extend to the 12.2% of the Chilean population living in rural areas, where exposure to sexual violence and access to services may differ. The two rounds also differ in age coverage (15–65 years in 2017; 15 years or older in 2020); all models adjust for age group and the oldest category is drawn from 2020 only, but the pooled prevalences average populations of different age structure. Finally, the ENVIF does not record gender identity, so the analysis cannot distinguish cisgender from transgender women. Nor does the survey collect race. It records self-identified belonging to one of the nine Indigenous peoples recognised under Chilean Law 19.253 at the time of the surveys, or to another Indigenous people, which is a distinct construct and does not capture Afro-descendant populations; migration status was available only in 2020. Although these data predate the pandemic and the 2024 ENVIF wave, they provide the most recent nationally representative estimates with municipal identifiers, which the 2024 wave did not release, and a pre-pandemic baseline against which subsequent changes can be assessed.

Three implications follow for practice and policy. First, prevention and clinical responses should treat IPSV, NPSA, and NPSH as distinct phenomena requiring different strategies: relational and life-course interventions for IPSV; territorial, urban-planning, and exposure-based approaches for NPSA and NPSH. Childhood sexual abuse, shared across the three forms, underscores trauma-informed screening and referral pathways in primary and mental health care. Second, systematic-review evidence links gender-inequitable norms and male sexual entitlement to perpetration, supporting prevention strategies that engage men.^31^ Third, these findings can refine implementation of Chile's GES 86: the profiles concentrating highest risk for sexual assault are younger women, women with childhood sexual abuse, women in high-dominance relationships, women whose partners are violent in other settings or were exposed to violence in childhood, and women in the Metropolitan Region, which allow the health system to prioritise outreach, early identification, and tailored support. The current GES 86 scope covers acute clinical care for sexual assault but not non-contact harassment; given NPSH's markedly higher prevalence and concentration among younger women, expanding territorial prevention and integrating gender-equality education in schools and workplaces remain a pending agenda. The 2017–2020 increase warrants close monitoring with the 2024 ENVIF round. For Latin America and the Caribbean, where nationally representative violence surveys often remain underused for disaggregated analysis, this study illustrates how separating partner, non-partner contact, and non-contact forms can generate more actionable evidence for health systems, urban prevention, and gender-equality policy.

## Contributors

FC: Conceptualisation, Methodology, Investigation, Formal analysis, Data curation, Funding acquisition, Writing—original draft, and Writing—review & editing. AF-G: Conceptualisation, Methodology, Formal analysis, Validation, Writing—review & editing, and Supervision. NL: Methodology, Formal analysis, Validation, Writing—review & editing, and Supervision. All authors critically reviewed the manuscript for important intellectual content, approved the final version for submission, and agree to be accountable for all aspects of the work. FC, AF-G and NL accessed and verified the underlying data. FC had the final responsibility for the decision to submit the manuscript.

## Data sharing statement

The ENVIF datasets analysed in this study are publicly available, fully anonymised, individual-level participant data released by the Subsecretaría de Prevención del Delito (Chile) at https://www.seguridadpublica.gov.cl/estadisticas/encuesta-nacional-de-violencia-intrafamiliar/. The full analytic code, a two-file reproducible pipeline covering variable construction, multilevel model specifications and all sensitivity analyses, together with the derived municipal context datasets, is publicly available on Figshare under a CC BY 4.0 licence (https://doi.org/10.6084/m9.figshare.33273933).

## Use of generative AI

During the preparation of this Article, the authors used Claude (Anthropic; model Claude Opus 4.7, accessed via the claude.ai web interface and the Claude desktop application between May and September, 2026) to assist with language editing and formatting of the English manuscript, and with the review and documentation of the R analysis code. The tool was not used to produce scientific insights, analyse or interpret the data, draw scientific conclusions, conduct the literature search, or generate references. All AI-assisted text was reviewed and edited by the authors, who take full responsibility for the accuracy, originality, and integrity of the published content.

## Declaration of interests

All authors declare no competing interests.
