# Supplementary material for: Social determinants of partner and non-partner sexual violence against women in Chile: a population-based multilevel analysis
Source: Lancet Reg Health Am. 2026 Oct 8;64:101656. doi: 10.1016/j.lana.2026.101656 (PMC13677301; doi:10.1016/j.lana.2026.101656)
Supplement: Supplementary file 1 [file mmc1.pdf]

Supplementary Material

Social determinants of partner and non-partner sexual violence against women in Chile: a population-based multilevel analysis

Crispi F, Fuentes-García A, Libuy N

Contents

Supplementary Table 1. Dependent variable components
Supplementary Table 2. Independent variables included in the analysis
Supplementary Table 3. Items composing the Individual Gender Index
Supplementary Table 4. Items composing the Dominant Relationship indicator
Supplementary Table 5. Missing data in partner-level covariates
Supplementary Table 6. Pearson correlation matrix
Supplementary Table 7. Variance inflation factors
Supplementary Table 8. Variance components, variance partition coefficient confidence intervals and quadrature
Supplementary Table 9. Missing data in the full pooled sample
Supplementary Table 10. Women included in and excluded from the IPSV model
Supplementary Table 11. IPSV fully adjusted model: complete case versus multiple imputation
Supplementary Table 12. Bivariate comparisons by IPSV status
Supplementary Table 13. Bivariate comparisons by NPSA status
Supplementary Table 14. Bivariate comparisons by NPSH status
Supplementary Table 15. Survey-weighted multilevel model for IPSV
Supplementary Table 16. Survey-weighted multilevel model for NPSA
Supplementary Table 17. Survey-weighted multilevel model for NPSH
Supplementary Table 18. Single-level logistic model for IPSV
Supplementary Table 19. Single-level logistic model for NPSA
Supplementary Table 20. Single-level logistic model for NPSH
Supplementary Table 21. Fully adjusted models estimated separately by wave
Supplementary Table 22. Likelihood-ratio tests for survey-wave interactions
Supplementary Table 23. IPSV fully adjusted model restricted to the 2020 wave
Supplementary Table 24. Events per variable and Firth penalised likelihood
Supplementary Table 25. IPSV under the two item definitions (5 harmonised items vs 8 items)
Supplementary Figure 1. Participant flow, ENVIF 2017 and 2020
Supplementary Figure 2. Past-12-month prevalence by age group and region

Supplementary Table 1. Dependent variable components

Outcome	Setting / question stem	Items
IPSV intimate partner sexual violence	Thinking of your different past and current intimate relationships, has your husband, cohabiting partner, or partner at that time ever... (2017: Section P73; 2020: Section XII, P132)	a) Forced you to have sexual intercourse when you did not want to.
IPSV intimate partner sexual violence	Thinking of your different past and current intimate relationships, has your husband, cohabiting partner, or partner at that time ever... (2017: Section P73; 2020: Section XII, P132)	b) Physically forced you to have sexual intercourse when you did not want to.
IPSV intimate partner sexual violence	Thinking of your different past and current intimate relationships, has your husband, cohabiting partner, or partner at that time ever... (2017: Section P73; 2020: Section XII, P132)	c) Forced you to perform a sexual act that you found humiliating or degrading.
IPSV intimate partner sexual violence	Thinking of your different past and current intimate relationships, has your husband, cohabiting partner, or partner at that time ever... (2017: Section P73; 2020: Section XII, P132)	d) Threatened to withhold household money if you did not have sex or engage in a sexual act when he/she wanted.
IPSV intimate partner sexual violence	Thinking of your different past and current intimate relationships, has your husband, cohabiting partner, or partner at that time ever... (2017: Section P73; 2020: Section XII, P132)	e) Forced you to stop using a method to prevent pregnancy.
IPSV intimate partner sexual violence	Thinking of your different past and current intimate relationships, has your husband, cohabiting partner, or partner at that time ever... (2017: Section P73; 2020: Section XII, P132)	f) Forced you to watch pornographic videos or films without your consent. (2020 only)
IPSV intimate partner sexual violence	Thinking of your different past and current intimate relationships, has your husband, cohabiting partner, or partner at that time ever... (2017: Section P73; 2020: Section XII, P132)	g) Forced you not to protect yourself sexually. (2020 only)
IPSV intimate partner sexual violence	Thinking of your different past and current intimate relationships, has your husband, cohabiting partner, or partner at that time ever... (2017: Section P73; 2020: Section XII, P132)	h) Forced you to record sexual content or to record yourself performing sexual acts. (2020 only)
NPSA non-partner sexual assault	Public spaces: In public places such as the street, transportation, entertainment venues, parks, churches, or health services, has it ever happened to you that someone known or unknown (not considering family members or a partner)... (P70)	3. Touched your body without your consent?
NPSA non-partner sexual assault	Public spaces: In public places such as the street, transportation, entertainment venues, parks, churches, or health services, has it ever happened to you that someone known or unknown (not considering family members or a partner)... (P70)	4. Forced you to have sexual relations or tried to force you to have sexual relations?
NPSA non-partner sexual assault	Public spaces: In public places such as the street, transportation, entertainment venues, parks, churches, or health services, has it ever happened to you that someone known or unknown (not considering family members or a partner)... (P70)	5. Forced you to perform sexual acts or to have sexual relations in exchange for money or other benefits?
NPSA non-partner sexual assault	Workplace: In your working life, has any boss/manager, director/supervisor, or co-worker... (P80)	e. Touched your body without your consent?
NPSA non-partner sexual assault	Workplace: In your working life, has any boss/manager, director/supervisor, or co-worker... (P80)	f. Forced you to have sexual relations or tried to force you to have sexual relations?
NPSA non-partner sexual assault	Educational settings: During your student life, has any authority figure (teacher, principal, etc.), administrative staff, or fellow student... (P90)	6. Touched your body without your consent?
NPSA non-partner sexual assault	Educational settings: During your student life, has any authority figure (teacher, principal, etc.), administrative staff, or fellow student... (P90)	7. Forced you to have sexual relations or tried to force you to have sexual relations?
NPSA non-partner sexual assault	Educational settings: During your student life, has any authority figure (teacher, principal, etc.), administrative staff, or fellow student... (P90)	8. Forced you to perform sexual acts or to have sexual relations in exchange for money or other benefits?
NPSH non-partner sexual harassment	Public spaces: In public places such as the street, transportation, entertainment venues, parks, churches, or health services, has it ever happened to you that someone known or unknown (not considering family members or a partner)... (P70)	1. Offended or denigrated you with obscene sexual phrases or words because you are a woman?
NPSH non-partner sexual harassment	Public spaces: In public places such as the street, transportation, entertainment venues, parks, churches, or health services, has it ever happened to you that someone known or unknown (not considering family members or a partner)... (P70)	2. Made you feel afraid of suffering a sexual attack or assault?

Outcome	Setting / question stem	Items
NPSH non-partner sexual harassment	Workplace: In your working life, has any boss/manager, director/supervisor, or co-worker... (P80)	c. Made sexual advances or propositions to you in exchange for improvements at work?
NPSH non-partner sexual harassment	Workplace: In your working life, has any boss/manager, director/supervisor, or co-worker... (P80)	d. Retaliated against you for refusing such advances?
NPSH non-partner sexual harassment	Educational settings: During your student life, has any authority figure (teacher, principal, etc.), administrative staff, or fellow student... (P90)	4. Made sexual advances or propositions to you in exchange for improvements in your grades or qualifications?
NPSH non-partner sexual harassment	Educational settings: During your student life, has any authority figure (teacher, principal, etc.), administrative staff, or fellow student... (P90)	5. Retaliated against you for refusing such advances?

IPSV = intimate partner sexual violence; NPSA = non-partner sexual assault (non-consensual physical sexual contact by someone other than a partner, including coerced or transactional sexual acts); NPSH = non-partner sexual harassment (sexual conduct without physical contact by someone other than a partner). Every module has a two-step structure: the stems shown are lifetime screening questions, and each affirmative answer is followed by a filter item recording whether the event occurred in the previous 12 months. All outcomes are defined on that filter, and a woman is classified as exposed if she answered "yes" to it for at least one component item. The filter items are, for intimate partner sexual violence, "Did this happen during the last 12 months?" (2017 P73.B; 2020 P132.B), and for the non-partner modules "Did this episode occur during the last 12 months?" in public spaces (2017 P94; 2020 P72), the workplace (2017 P102; 2020 P82) and educational settings (2017 P87; 2020 P94). Item numbering is not parallel across waves: P94 denotes the public-space filter in 2017 and the educational filter in 2020. In 2020 the educational-module filter was administered only to women who reported being currently studying; no equivalent restriction applied in 2017. This makes the 2020 educational contribution to NPSA and NPSH conservative relative to 2017. The primary IPSV definition uses the five items asked identically or near-identically in both waves (a to e; 2017 P73.a-e, 2020 P132.a-e; item d reads "if you did not have sex" in 2017 and "if you did not have sex or engage in a sexual act" in 2020, the wording shown above); items f, g and h were added in 2020 and enter only the eight-item sensitivity definition (Supplementary Table 25). IPSV items apply to women who reported a partner in the previous 12 months; NPSA and NPSH items apply to all women in the sample.

Supplementary Table 2. Independent variables included in the analysis

Variable	Type and categories	Description and source
INDIVIDUAL LEVEL		
Age	Categorical (7 groups, ENVIF original): 1 = 15-18; 2 = 19-25; 3 = 26-35; 4 = 36-45; 5 = 46-55; 6 = 56-65; 7 = 66 and older	Self-reported age in years, categorised into the seven age groups used in the original ENVIF questionnaire.
Educational attainment	Categorical (4 levels): 0 = No completed studies; 1 = Primary completed; 2 = Secondary completed; 3 = Tertiary (university) completed	Highest educational level completed by the respondent at the time of the survey.
Socioeconomic status (SES)	Categorical (3 levels): 1 = Low; 2 = Middle; 3 = High	Socioeconomic group (GSE) as supplied with the ENVIF datasets, derived by the survey contractor using the AIM Chile 2019 classification, which combines the respondent's education, the occupation of the main household earner, household size and household income bracket (https://aimchile.cl/gse-chile/). The AIM groups were collapsed into three levels (low, middle, high).
Marital status	Dichotomous: 0 = Single, separated, divorced, annulled or widowed; 1 = Married or in a registered civil union (acuerdo de unión civil)	Self-reported legal marital status at the time of the survey (2017 P8; 2020 P3). Cohabitation without a registered union is not recorded separately and is classified as single.
Indigenous identity	Dichotomous: 1 = Yes; 0 = No	Based on the question: "Do you identify with any of the Indigenous peoples recognised by Chilean law?" (nine recognised groups). Women who named a people other than the nine listed (option "other"; 46 in 2017 and 48 in 2020) are counted as Indigenous.
Disability status	Dichotomous: 1 = Yes; 0 = No	Coded 1 when the respondent reports at least one of the following permanent conditions: physical or mobility difficulty; muteness or speaking difficulty; psychiatric difficulty; intellectual or mental difficulty; deafness or hearing difficulty (even when using hearing aids); blindness or seeing difficulty (even when using glasses).
Own income	Dichotomous: 1 = Yes; 0 = No	Based on the question: "Do you generate your own income, whether from formal or informal work?" Lack of own income is interpreted as greater economic dependence.
Social participation	Dichotomous: 1 = Participates at least once per month; 0 = Does not participate	Indicator of potential social isolation. Captures regular participation in one or more social organisations: neighbourhood council, women's organisation, sports/cultural/recreational organisation, political party or trade union, religious organisation, cooperative or production group, ethnic or Indigenous organisation, parent-teacher association or school activities, volunteer group, neighbourhood committee, or other organisation.
Childhood sexual abuse	Dichotomous: 1 = Yes; 0 = No	Constructed from a single item asking whether anyone touched the respondent sexually or forced her to perform a sexual act against her will before the age of 14, perpetrated by a family member, partner, or ex-partner. Used as an individual-level risk factor for adult victimisation. The 2017 and 2020 items are identical in content; the 2020 wording adds illustrative examples (sexual touching or kissing, simulation of a sexual act, exposure to or recording of pornographic material).
Individual Gender Index	Continuous (z-score, standardised within each survey year)	Composite index built from 14 items in 2017 and 13 items in 2020 capturing attitudes toward gender roles, relationship power dynamics, and the acceptability of violence. Each item uses a 5-point Likert scale (1 = strongly disagree to 5 = strongly agree). All items were harmonised so that 1 represents more egalitarian views and 5 more traditional views. Item scores were summed and the global score standardised within year (z-score). Higher z-scores indicate more traditional gender attitudes. Internal consistency: Cronbach alpha 0·90 in 2017; 0·75 in 2020. Full item list in Supplementary Table 3.
RELATIONSHIP LEVEL (IPSV models only)		
Dominant relationship indicator	Dichotomous: 1 = At least one controlling behaviour reported; 0 = None reported	Global indicator of control within the intimate relationship, derived from a 10-item (2017) and 11-item (2020) battery on controlling behaviours, summarised as eight merged items. Coded 1 when the respondent reports at least one of these behaviours by her partner. Full item list in Supplementary Table 4.
Partner's childhood exposure to violence	Dichotomous: 1 = Yes; 0 = No	Coded 1 when the respondent reports that her current (or most recent) partner experienced or witnessed violence in his childhood. Responses of "don't know" coded as no known exposure (0) and "no answer" as missing, identically in both rounds (2017 P35 and P36; 2020 P39 and P40, with parallel items for a former partner).

Variable	Type and categories	Description and source
Partner violent in other settings	Dichotomous: 1 = Yes; 0 = No	Based on the current-partner item in each wave, with a parallel item for a former partner (2017 P33 and P43; 2020 P37 and P51). 2017 (P33): "Does your current husband/partner or boyfriend/girlfriend assault other people in the street, argue aggressively, or take part in street fights with other people (people outside the family)?" 2020 (P37): "To your knowledge, has your current partner (husband, partner or boyfriend/girlfriend) caused harm to other people in public spaces (i.e., attacking, humiliating, insulting or belittling) (people outside the family)?" The 2020 wording is broader, as it includes non-physical harm.
COMMUNITY LEVEL (municipal)		
Municipal poverty rate	Continuous (proportion of population in income poverty)	Municipal income-poverty rate estimated with small area estimation (SAE) methods by the Ministry of Social Development from the National Socioeconomic Characterisation Survey (CASEN). ENVIF 2017 matched to CASEN 2017 estimates; ENVIF 2020 matched to CASEN 2020 estimates (September 2022 revision).
Municipal crime rate	Continuous (cases per 100 000 inhabitants)	Municipal rate of crimes of greater social connotation (delitos de mayor connotación social, DMCS) per 100 000 inhabitants, from the Centro de Estudios y Análisis del Delito (CEAD), Subsecretaria de Prevención del Delito. ENVIF 2017 matched to 2017 rates; ENVIF 2020 matched to 2019 rates (closest pre-fieldwork year), under an identical definition in both waves.
Municipal gender norms	Continuous (z-score)	Municipal-level mean of the Individual Gender Index across all respondents in each municipality, standardised (z-score). Higher values indicate more traditional gender norms at the municipal level.
CONTEXTUAL / SOCIETAL		
Metropolitan Region	Dichotomous: 1 = Yes; 0 = No	16 regions were collapsed into a dummy variable.
Year of survey	Dichotomous: 0 = 2017; 1 = 2020	Indicator of the survey wave, included as a fixed effect to account for temporal differences between rounds.

All variables refer to data drawn from the 2017 and 2020 rounds of the Encuesta Nacional de Violencia Intrafamiliar contra la Mujer y en Otros Espacios (ENVIF), unless otherwise specified. IPSV = intimate partner sexual violence.

Supplementary Table 3. Items composing the Individual Gender Index

Introductory question: "To what extent do you agree or disagree with the following statements?" Each item uses a 5-point Likert scale (1 = strongly disagree to 5 = strongly agree). All items were harmonised so that 1 indicates a more egalitarian view and 5 a more traditional view. Item scores were summed and standardised within each year (z-score). Higher values indicate more traditional attitudes.

#	Item	Wave
1	It is more appropriate for the man to be recognised as head of household rather than the woman.	2017, 2020
2	The man should be responsible for family and household expenses.	2017, 2020
3	The woman should take care of the children instead of the man.	2017, 2020
4	Doing household chores (cleaning, washing, ironing, cooking) is a task more appropriate for women than for men.	2017, 2020
5	A wife/partner should not contradict her husband/partner's opinion.	2017, 2020
6	A woman may participate in a social activity even if she does not have her husband/partner's approval.*	2017, 2020
7	A woman may choose her friends even if her husband does not like it.*	2017, 2020
8	A woman's clothing and makeup should be approved by her husband/partner.	2017, 2020
9	A woman should have sexual relations with her husband/partner, even if she does not want to.	2017, 2020
10	A woman should avoid dressing provocatively so as not to be a victim of harassment.	2017, 2020
11	Women should accept mistreatment for the good of the family and the children.	2017, 2020
12	If there is hitting or mistreatment at home, it is a matter that should be resolved within the family.	2017, 2020
13	It is acceptable for a man to assault his partner in the event of infidelity.	2017, 2020
14	If a couple cannot agree on something, the final decision should be made by the man if he is the one who provides for the household.	2017 only

*Wording shown is that of the 2020 questionnaire; the 14 items of the 2017 questionnaire are equivalent in content to items 1 to 14 but several differ in phrasing (for example, item 10 read "If a woman is harassed, she is probably responsible for wearing provocative clothing" in 2017). *Items 6 and 7 were reverse-coded prior to summation in 2020, where they were worded in the egalitarian direction; the corresponding 2017 items were worded in the traditional direction and were not reversed. Higher values consistently indicate more traditional attitudes. Source: ENVIF 2017 and 2020.*

Supplementary Table 4. Items composing the Dominant Relationship indicator

The Dominant Relationship indicator is a global measure of control within the intimate relationship, derived from the controlling-behaviour battery of each questionnaire (10 items in 2017, P64; 11 items in 2020, P115), summarised below as eight merged items. The variable is coded 1 when the respondent reports having experienced at least one of these behaviours by her partner, and 0 otherwise.

#	Item
1	Has your partner ever tried to prevent you from seeing your friends?
2	Has your partner ever tried to limit your contact with your close family?
3	Has your partner ever told you that everything you do is wrong, that you are clumsy or useless?
4	Has your partner ever become angry if you spoke with other people by mobile phone or social networks?
5	Does your partner often suspect that you are unfaithful?
6	Does your partner expect you to ask for permission before seeking health care for yourself?
7	Does your partner control or check your mobile phone, WhatsApp or other applications, or ask you for your passwords?
8	Does your partner insist on being included in your social media profiles, or forbid you from using these applications?

Source: ENVIF 2017 and 2020. Wording shown is that of the 2020 questionnaire. Item 3 was asked only in 2020. Item 4 read "Has your partner ever become angry if you spoke with another man?" in 2017. The 2017 questionnaire additionally asked whether the partner insisted on knowing with whom the respondent was at all times, and the 2020 questionnaire added an item on asking for passwords, merged here into item 7.

Supplementary Table 5. Missing data in partner-level covariates

Women who reported a partner in the last 12 months, ENVIF 2017 and 2020

Variable	N total	Pooled 2017-2020		ENVIF 2017		ENVIF 2020	
		n (pooled)	% (pooled)	n (2017)	% (2017)	n (2020)	% (2020)
Dominant relationship	9 969	595	6·0	288	5·9	307	6·0
Partner: violence in childhood	9 969	43	0·4	25	0·5	18	0·4
Partner: violent in other settings	9 969	202	2·0	68	1·4	134	2·6
Partner: high alcohol consumption	9 969	2 918	29·3	45	0·9	2 873	56·2
Partner: age difference	9 969	667	6·7	331	6·8	336	6·6
Partner: education	9 969	3 826	38·4	325	6·7	3 501	68·5

Partner alcohol consumption, partner education and partner age difference were excluded a priori from the multilevel models because of item administration problems in the 2020 round. Responses of "don't know" on the partner's childhood exposure to violence are coded as no known exposure in both rounds. The partner items were administered with a different response format in the two rounds, which motivates the 2020-only sensitivity analysis (Supplementary Table 23); the multiple-imputation analysis (Supplementary Table 11) addresses the residual missingness. ENVIF = Encuesta Nacional de Violencia Intrafamiliar y en Otros Espacios (National Survey on Violence in the Family and Other Settings).

Supplementary Table 6. Pearson correlation matrix

	Metropolitan Region	Age (years)	Education	Socioeconomic level	Married or in a civil union	Social participation	Indigenous identity	Disability	Childhood sexual abuse	Individual Gender Index	Partner: violence in childhood	Partner: violent in other settings	Dominant relationship	Own income	Municipal Gender Index	Municipal poverty rate	Municipal crime rate
Metropolitan Region	1·00	0·03	0·02	0·07	0·01	-0·08	-0·07	0·00	0·03	-0·01	0·03	0·01	0·05	0·01	-0·01	-0·22	0·17
Age (years)	0·03	1·00	-0·30	-0·09	0·26	0·11	-0·07	0·26	-0·02	0·29	0·03	-0·03	-0·08	-0·07	0·07	0·03	-0·02
Education	0·02	-0·30	1·00	0·43	-0·03	0·00	-0·04	-0·18	0·00	-0·30	-0·06	-0·03	-0·03	0·24	-0·09	-0·08	0·13
Socioeconomic level	0·07	-0·09	0·43	1·00	0·06	-0·03	-0·05	-0·13	-0·04	-0·21	-0·07	-0·03	-0·07	0·13	-0·16	-0·17	0·12
Married or in a civil union	0·01	0·26	-0·03	0·06	1·00	0·09	-0·04	0·00	-0·02	0·10	0·03	-0·08	-0·09	-0·12	0·00	-0·01	-0·02
Social participation	-0·08	0·11	0·00	-0·03	0·09	1·00	0·08	0·05	0·04	0·08	0·07	-0·01	0·02	-0·01	0·08	0·08	-0·06
Indigenous identity	-0·07	-0·07	-0·04	-0·05	-0·04	0·08	1·00	0·03	0·04	0·01	0·06	0·00	0·01	0·03	0·05	-0·02	-0·07
Disability	0·00	0·26	-0·18	-0·13	0·00	0·05	0·03	1·00	0·05	0·16	0·06	0·02	0·03	-0·10	0·10	0·06	-0·03
Childhood sexual abuse	0·03	-0·02	0·00	-0·04	-0·02	0·04	0·04	0·05	1·00	-0·02	0·12	0·04	0·14	0·05	0·05	0·04	0·01
Individual Gender Index	-0·01	0·29	-0·30	-0·21	0·10	0·08	0·01	0·16	-0·02	1·00	0·03	-0·01	0·03	-0·13	0·44	0·15	-0·08
Partner: violence in childhood	0·03	0·03	-0·06	-0·07	0·03	0·07	0·06	0·06	0·12	0·03	1·00	0·16	0·17	0·05	0·05	0·03	-0·03
Partner: violent in other settings	0·01	-0·03	-0·03	-0·03	-0·08	-0·01	0·00	0·02	0·04	-0·01	0·16	1·00	0·15	0·01	-0·02	0·01	0·01
Dominant relationship	0·05	-0·08	-0·03	-0·07	-0·09	0·02	0·01	0·03	0·14	0·03	0·17	0·15	1·00	0·03	0·02	0·05	0·00
Own income	0·01	-0·07	0·24	0·13	-0·12	-0·01	0·03	-0·10	0·05	-0·13	0·05	0·01	0·03	1·00	0·00	-0·02	0·01
Municipal Gender Index	-0·01	0·07	-0·09	-0·16	0·00	0·08	0·05	0·10	0·05	0·44	0·05	-0·02	0·02	0·00	1·00	0·33	-0·19
Municipal poverty rate	-0·22	0·03	-0·08	-0·17	-0·01	0·08	-0·02	0·06	0·04	0·15	0·03	0·01	0·05	-0·02	0·33	1·00	-0·22
Municipal crime rate	0·17	-0·02	0·13	0·12	-0·02	-0·06	-0·07	-0·03	0·01	-0·08	-0·03	0·01	0·00	0·01	-0·19	-0·22	1·00

Unweighted product-moment correlations; ordinal variables (education, socioeconomic level, age group) entered as numeric codes and binary variables as 0/1. Descriptive only. Multicollinearity in the fitted models is assessed with variance inflation factors (Supplementary Table 7).

Supplementary Table 7. Variance inflation factors

Fully adjusted models

Term	VIF	Tolerance
**IPSV**		
Age group	1·96	0·510
Education	1·63	0·613
Married or in a civil union	1·44	0·695
Municipal Gender Index (z)	1·34	0·748
Socioeconomic level	1·33	0·754
Individual Gender Index (z)	1·33	0·750
Municipal poverty rate (z)	1·29	0·775
Survey wave	1·15	0·869
Dominant relationship	1·13	0·882
Partner: violence in childhood	1·13	0·886
Partner: violent in other settings	1·11	0·903
Own income	1·11	0·897
Disability	1·10	0·908
Municipal crime rate (z)	1·09	0·920
Metropolitan Region	1·08	0·930
Childhood sexual abuse	1·07	0·932
Social participation	1·07	0·938
Indigenous identity	1·04	0·959
**NPSA**		
Age group	2·03	0·493
Education	1·81	0·553
Municipal poverty rate (z)	1·46	0·684
Municipal Gender Index (z)	1·37	0·730
Individual Gender Index (z)	1·34	0·744
Socioeconomic level	1·31	0·761
Survey wave	1·21	0·828
Own income	1·20	0·835
Metropolitan Region	1·19	0·842
Married or in a civil union	1·18	0·847
Municipal crime rate (z)	1·15	0·869
Disability	1·09	0·914
Childhood sexual abuse	1·06	0·944
Social participation	1·05	0·956
Indigenous identity	1·03	0·967
**NPSH**		
Age group	2·05	0·487
Education	1·85	0·542
Municipal poverty rate (z)	1·49	0·669
Socioeconomic level	1·31	0·763
Municipal Gender Index (z)	1·30	0·767
Individual Gender Index (z)	1·25	0·799
Survey wave	1·22	0·819
Married or in a civil union	1·21	0·829
Own income	1·21	0·827
Metropolitan Region	1·18	0·844
Municipal crime rate (z)	1·18	0·845

Term	VIF	Tolerance
Disability	1·08	0·923
Childhood sexual abuse	1·04	0·960
Social participation	1·04	0·960
Indigenous identity	1·02	0·978

Variance inflation factors are the primary multicollinearity diagnostic. The correlation matrix in Supplementary Table 6 is retained as a descriptive summary only, because most covariates are binary or categorical and product-moment correlations do not index linear dependence for them. VIF = variance inflation factor; IPSV = intimate partner sexual violence; NPSA = non-partner sexual assault; NPSH = non-partner sexual harassment.

Supplementary Table 8a. Unrounded municipality variance components

Model	Level-2 variance	Level-2 SD	VPC (%)	Singular fit
**IPSV**				
M0	0·000e+00	0·000e+00	0·0000	Yes
M1	0·000e+00	0·000e+00	0·0000	Yes
M2	0·000e+00	0·000e+00	0·0000	Yes
M3	6·296e-18	2·509e-09	0·0000	Yes
**NPSA**				
M0	2·981e-01	5·459e-01	8·3071	No
M1	3·016e-01	5·492e-01	8·3984	No
M2	1·213e-01	3·482e-01	3·5546	No
M3	1·133e-01	3·365e-01	3·3281	No
**NPSH**				
M0	2·964e-01	5·445e-01	8·2661	No
M1	2·948e-01	5·429e-01	8·2227	No
M2	1·428e-01	3·779e-01	4·1593	No
M3	1·494e-01	3·866e-01	4·3448	No

Variance components are shown unrounded so that a boundary estimate can be distinguished from a small non-zero value rounded to zero. Where lme4 reports a singular fit, the estimated between-municipality variance has converged to the boundary of the parameter space, and the variance partition coefficient should be read as approximately zero rather than exactly zero. M0 = empty model (municipality random intercept only); M1 = M0 plus individual-level (and, for IPSV, relationship-level) covariates; M2 = M0 plus contextual covariates (Metropolitan Region and municipal-level variables) and survey wave; M3 = full model with all covariates. SD = standard deviation; VPC = variance partition coefficient; IPSV = intimate partner sexual violence; NPSA = non-partner sexual assault; NPSH = non-partner sexual harassment.

Supplementary Table 8b. Confidence intervals for the variance partition coefficient

Model	Method	VPC (%)	95% CI
**IPSV**			
M0	profile likelihood	0·00	0·00 to 3·18
M3	profile likelihood	0·00	0·00 to 2·57
**NPSA**			
M0	profile likelihood	8·31	2·70 to 16·31
M3	profile likelihood	3·33	0·00 to 9·52
**NPSH**			
M0	profile likelihood	8·27	5·18 to 12·56
M3	profile likelihood	4·34	2·17 to 7·62

Profile-likelihood intervals. Where the level-2 variance is at its boundary, the interval is bounded below by zero and the point estimate is highly sensitive to specification. M0 = empty model (municipality random intercept only); M3 = fully adjusted model with all covariates. VPC = variance partition coefficient; CI = confidence interval; IPSV = intimate partner sexual violence; NPSA = non-partner sexual assault; NPSH = non-partner sexual harassment.

Supplementary Table 8c. IPSV fully adjusted model: Laplace versus adaptive Gauss-Hermite quadrature

nAGQ = 10

Variables	Laplace	Adaptive quadrature	Difference (%)
Age group: 19-25	0·84 (0·36 to 1·97)	0·84 (0·36 to 1·97)	0·00
Age group: 26-35	0·46 (0·20 to 1·07)	0·46 (0·20 to 1·07)	0·00
Age group: 36-45	0·51 (0·22 to 1·20)	0·51 (0·22 to 1·20)	0·00
Age group: 46-55	0·45 (0·19 to 1·06)	0·45 (0·19 to 1·06)	0·00
Age group: 56-65	0·47 (0·20 to 1·13)	0·47 (0·20 to 1·13)	0·00
Age group: 66 or more	0·09 (0·02 to 0·46)	0·09 (0·02 to 0·46)	0·00
Socioeconomic level: Medium	0·90 (0·65 to 1·26)	0·90 (0·65 to 1·26)	0·00
Socioeconomic level: High	1·35 (0·67 to 2·69)	1·35 (0·67 to 2·69)	0·00
Education: Primary	1·15 (0·67 to 1·99)	1·15 (0·67 to 1·99)	0·00
Education: Secondary	0·94 (0·54 to 1·64)	0·94 (0·54 to 1·64)	0·00
Education: Tertiary	0·56 (0·28 to 1·11)	0·56 (0·28 to 1·11)	0·00

Variables	Laplace	Adaptive quadrature	Difference (%)
Married or in a civil union	1·21 (0·85 to 1·72)	1·21 (0·85 to 1·72)	0·00
Metropolitan Region	0·61 (0·38 to 0·96)	0·61 (0·38 to 0·96)	0·00
Indigenous identity	1·08 (0·71 to 1·62)	1·08 (0·71 to 1·62)	0·00
Disability	1·37 (0·85 to 2·23)	1·37 (0·85 to 2·23)	0·00
Childhood sexual abuse	2·05 (1·45 to 2·89)	2·05 (1·45 to 2·89)	0·00
Individual Gender Index (z)	1·04 (0·88 to 1·24)	1·04 (0·88 to 1·24)	0·00
Own income	0·84 (0·61 to 1·14)	0·84 (0·61 to 1·14)	0·00
Social participation	1·07 (0·79 to 1·45)	1·07 (0·79 to 1·45)	0·00
Dominant relationship	6·40 (4·68 to 8·76)	6·40 (4·68 to 8·76)	0·00
Partner: violence in childhood	1·60 (1·17 to 2·18)	1·60 (1·17 to 2·18)	0·00
Partner: violent in other settings	2·79 (1·75 to 4·43)	2·79 (1·75 to 4·43)	0·00
Municipal Gender Index (z)	1·00 (0·83 to 1·21)	1·00 (0·83 to 1·21)	0·00
Municipal poverty rate (z)	0·98 (0·82 to 1·16)	0·98 (0·82 to 1·16)	0·00
Municipal crime rate (z)	1·02 (0·86 to 1·21)	1·02 (0·86 to 1·21)	0·00
Survey wave: 2020 (ref: 2017)	1·41 (1·02 to 1·95)	1·41 (1·02 to 1·95)	0·00

The primary models are fitted by maximum likelihood with the Laplace approximation (nAGQ = 1). The low-prevalence IPSV model is refitted with adaptive quadrature to confirm that the fixed effects are not sensitive to the approximation. nAGQ = number of adaptive Gauss-Hermite quadrature points. OR = odds ratio; CI = confidence interval; ref = reference category; (z) = z-standardised variable (Individual Gender Index within survey wave; municipal covariates on the pooled analytic sample); IPSV = intimate partner sexual violence.

Supplementary Table 9. Missing data in the full pooled sample

ENVIF 2017 and 2020

Variable	N total	Pooled 2017-2020		ENVIF 2017		ENVIF 2020	
		n (pooled)	% (pooled)	n (2017)	% (2017)	n (2020)	% (2020)
Intimate partner sexual violence	14 559	2 256	15·5	2 256	33·1	0	0·0
Non-partner sexual assault	14 559	0	0·0	0	0·0	0	0·0
Non-partner sexual harassment	14 559	0	0·0	0	0·0	0	0·0
Age (years)	14 559	0	0·0	0	0·0	0	0·0
Age group	14 559	0	0·0	0	0·0	0	0·0
Socioeconomic level	14 559	0	0·0	0	0·0	0	0·0
Education	14 559	22	0·2	10	0·1	12	0·2
Married or in a civil union	14 559	11	0·1	6	0·1	5	0·1
Metropolitan Region	14 559	0	0·0	0	0·0	0	0·0
Indigenous identity	14 559	344	2·4	273	4·0	71	0·9
Disability	14 559	220	1·5	199	2·9	21	0·3
Childhood sexual abuse	14 559	142	1·0	60	0·9	82	1·1
Individual Gender Index	14 559	690	4·7	317	4·6	373	4·8
Own income	14 559	142	1·0	40	0·6	102	1·3
Social participation	14 559	234	1·6	144	2·1	90	1·2
Municipal Gender Index	14 559	0	0·0	0	0·0	0	0·0
Municipal poverty rate	14 559	0	0·0	0	0·0	0	0·0
Municipal crime rate	14 559	0	0·0	0	0·0	0	0·0

Partner-level covariates are reported separately in Supplementary Table 5, where the denominator is women with a partner in the last 12 months. ENVIF = Encuesta Nacional de Violencia Intrafamiliar y en Otros Espacios (National Survey on Violence in the Family and Other Settings).

Supplementary Table 10. Women included in and excluded from the IPSV model

Denominator: women with a partner in the last 12 months

Variable	Level	Excluded (n = 1 634)	Included (n = 8 335)	p-value
Age (years)	mean (SD)	41·8 (16·1)	43·8 (14·3)	<0·0001
Age group	15-18	5·4	1·8	<0·0001
Age group	19-25	15·3	9·4	<0·0001
Age group	26-35	18·5	21·7	<0·0001
Age group	36-45	18·7	20·8	<0·0001
Age group	46-55	18·2	21·6	<0·0001
Age group	56-65	18·7	20·8	<0·0001
Age group	66 or more	5·3	3·9	<0·0001
Education	None	10·8	9·7	0·0008
Education	Primary	25·7	24·3	0·0008
Education	Secondary	44·2	42·1	0·0008
Education	Tertiary	19·3	23·9	0·0008
Socioeconomic level	Low	52·8	46·8	<0·0001
Socioeconomic level	Medium	41·9	45·3	<0·0001
Socioeconomic level	High	5·4	7·9	<0·0001
Survey wave	2017	51·3	48·2	0·021
Survey wave	2020	48·7	51·8	0·021
Metropolitan Region	No	85·6	84·4	0·24
Metropolitan Region	Yes	14·4	15·6	0·24
Indigenous identity	No	81·6	87·2	<0·0001
Indigenous identity	Yes	18·4	12·8	<0·0001
Disability	No	89·7	92·7	<0·0001
Disability	Yes	10·3	7·3	<0·0001
Childhood sexual abuse	No	87·7	89·0	0·13
Childhood sexual abuse	Yes	12·3	11·0	0·13
Intimate partner sexual violence	No	94·7	97·6	<0·0001
Intimate partner sexual violence	Yes	5·3	2·4	<0·0001

Unweighted comparisons. Continuous variables are compared with t-tests and categorical variables with chi-square tests. Percentages are column percentages within each group. SD = standard deviation; IPSV = intimate partner sexual violence.

Supplementary Table 11. IPSV fully adjusted model: complete case versus multiple imputation

Chained equations, m = 20 imputations

Variables	Complete case	Multiple imputation
Age group: 19-25	0·84 (0·36 to 1·97)	1·06 (0·51 to 2·20)
Age group: 26-35	0·46 (0·20 to 1·07)	0·48 (0·23 to 1·01)
Age group: 36-45	0·51 (0·22 to 1·20)	0·58 (0·27 to 1·23)
Age group: 46-55	0·45 (0·19 to 1·06)	0·46 (0·22 to 0·98)
Age group: 56-65	0·47 (0·20 to 1·13)	0·53 (0·25 to 1·15)
Age group: 66 or more	0·09 (0·02 to 0·46)	0·11 (0·03 to 0·42)
Socioeconomic level: Medium	0·90 (0·65 to 1·26)	0·89 (0·66 to 1·18)
Socioeconomic level: High	1·35 (0·67 to 2·69)	1·12 (0·60 to 2·08)
Education: Primary	1·15 (0·67 to 1·99)	0·84 (0·52 to 1·34)
Education: Secondary	0·94 (0·54 to 1·64)	0·77 (0·48 to 1·24)
Education: Tertiary	0·56 (0·28 to 1·11)	0·49 (0·27 to 0·88)
Married or in a civil union	1·21 (0·85 to 1·72)	1·19 (0·87 to 1·61)
Metropolitan Region	0·61 (0·38 to 0·96)	0·63 (0·42 to 0·95)
Indigenous identity	1·08 (0·71 to 1·62)	1·08 (0·76 to 1·54)
Disability	1·37 (0·85 to 2·23)	1·33 (0·87 to 2·03)
Childhood sexual abuse	2·05 (1·45 to 2·89)	2·26 (1·67 to 3·05)
Individual Gender Index (z)	1·04 (0·88 to 1·24)	0·97 (0·83 to 1·13)
Own income	0·84 (0·61 to 1·14)	0·91 (0·69 to 1·20)
Social participation	1·07 (0·79 to 1·45)	1·04 (0·79 to 1·35)
Dominant relationship	6·40 (4·68 to 8·76)	6·30 (4·71 to 8·43)
Partner: violence in childhood	1·60 (1·17 to 2·18)	1·56 (1·19 to 2·06)
Partner: violent in other settings	2·79 (1·75 to 4·43)	4·06 (2·82 to 5·85)
Municipal Gender Index (z)	1·00 (0·83 to 1·21)	1·00 (0·85 to 1·18)
Municipal poverty rate (z)	0·98 (0·82 to 1·16)	0·97 (0·83 to 1·14)
Municipal crime rate (z)	1·02 (0·86 to 1·21)	1·04 (0·89 to 1·21)
Survey wave: 2020 (ref: 2017)	1·41 (1·02 to 1·95)	1·52 (1·14 to 2·03)

OR (95% CI). Missingness in the retained covariates is below 5% except for dominant relationship (6·0%; Supplementary Table 5); data are assumed missing at random conditional on survey wave and covariates. The imputation model is single level; municipal contextual variables are included as predictors but no municipality random effect is imputed. The analysis model fitted to each completed dataset is the full two-level model. OR = odds ratio; CI = confidence interval; ref = reference category; (z) = z-standardised variable (Individual Gender Index within survey wave; municipal covariates on the pooled analytic sample); IPSV = intimate partner sexual violence.

Supplementary Table 12. Bivariate comparisons by IPSV status

Women with a partner in the last 12 months, ENVIF 2017 and 2020

Variables	Non-victim (n = 9 410)	Victim (n = 271)	Test	p-value
Age (years)	40·8	35·8	t = -3·9	0·0001
Age group			chi-sq = 75·3	<0·0001
15-18	3·9	8·8		
19-25	13·6	28·5		
26-35	24·4	17·2		
36-45	21·0	18·4		
46-55	19·2	15·1		
56-65	12·9	10·7		
66 or more	5·1	1·3		
Socioeconomic level			chi-sq = 11·8	0·095
Low	45·5	56·0		
Medium	45·9	36·9		
High	8·6	7·1		
Education			chi-sq = 13·8	0·098
None	8·2	11·2		
Primary	22·6	29·8		
Secondary	43·0	39·3		
Tertiary	26·2	19·8		
Married or in a civil union	50·0	37·8	chi-sq = 15·9	0·0042
Metropolitan Region	46·1	40·2	chi-sq = 3·8	0·23
Indigenous identity	9·0	14·9	chi-sq = 10·7	0·027
Disability	6·3	11·0	chi-sq = 9·9	0·027
Childhood sexual abuse	11·9	34·0	chi-sq = 117·2	<0·0001
Individual Gender Index	26·5	26·6	t = 0·1	0·90
Own income	59·2	61·4	chi-sq = 0·5	0·62
Social participation	39·1	39·4	chi-sq = 0·0	0·95
Dominant relationship	17·3	61·2	chi-sq = 281·0	<0·0001
Partner: violence in childhood	25·5	52·7	chi-sq = 101·6	<0·0001
Partner: violent in other settings	3·3	25·0	chi-sq = 318·7	<0·0001
Municipal Gender Index	26·9	27·3	t = 1·3	0·19
Municipal poverty rate	9·2	9·7	t = 1·1	0·25
Municipal crime rate	3 176	3 081	t = -1·0	0·33

Weighted means or percentages. Survey-adjusted t-tests for continuous variables and design-adjusted Rao-Scott chi-square tests for categorical variables. t = survey-adjusted t statistic; chi-sq = design-adjusted Rao-Scott chi-square statistic; IPSV = intimate partner sexual violence; ENVIF = Encuesta Nacional de Violencia Intrafamiliar y en Otros Espacios (National Survey on Violence in the Family and Other Settings).

Supplementary Table 13. Bivariate comparisons by NPSA status

Full pooled sample, ENVIF 2017 and 2020

Variables	Non-victim (n = 14 347)	Victim (n = 212)	Test	p-value
Age (years)	41·6	30·7	t = -9·5	<0·0001
Age group			chi-sq = 226·5	<0·0001
15-18	7·0	13·3		
19-25	13·7	37·9		
26-35	20·8	18·8		
36-45	18·3	13·7		
46-55	18·2	9·8		
56-65	14·2	5·6		
66 or more	7·8	0·8		
Socioeconomic level			chi-sq = 0·4	0·93
Low	48·0	48·9		
Medium	44·0	42·4		
High	8·0	8·7		
Education			chi-sq = 15·4	0·16
None	9·9	5·5		
Primary	24·0	21·6		
Secondary	41·3	50·3		
Tertiary	24·8	22·7		
Married or in a civil union	35·7	11·9	chi-sq = 89·0	<0·0001
Metropolitan Region	44·6	66·6	chi-sq = 69·5	<0·0001
Indigenous identity	9·1	11·7	chi-sq = 2·9	0·29
Disability	8·3	5·9	chi-sq = 2·6	0·31
Childhood sexual abuse	11·6	23·8	chi-sq = 50·1	<0·0001
Individual Gender Index	26·5	24·5	t = -2·9	0·0040
Own income	58·7	61·8	chi-sq = 1·4	0·51
Social participation	38·4	41·5	chi-sq = 1·4	0·50
Municipal Gender Index	27·0	26·9	t = -0·1	0·89
Municipal poverty rate	9·3	8·8	t = -1·3	0·18
Municipal crime rate	3 177	3 290	t = 0·8	0·40

Weighted means or percentages. Survey-adjusted t-tests for continuous variables and design-adjusted Rao-Scott chi-square tests for categorical variables. t = survey-adjusted t statistic; chi-sq = design-adjusted Rao-Scott chi-square statistic; NPSA = non-partner sexual assault; ENVIF = Encuesta Nacional de Violencia Intrafamiliar y en Otros Espacios (National Survey on Violence in the Family and Other Settings).

Supplementary Table 14. Bivariate comparisons by NPSH status

Full pooled sample, ENVIF 2017 and 2020

Variables	Non-victim (n = 13 455)	Victim (n = 1 104)	Test	p-value
Age (years)	42·9	29·0	t = -26·6	<0·0001
Age group			chi-sq = 1155·4	<0·0001
15-18	5·8	17·9		
19-25	12·0	32·4		
26-35	20·1	25·9		
36-45	19·0	11·4		
46-55	19·2	8·2		
56-65	15·4	3·0		
66 or more	8·5	1·1		
Socioeconomic level			chi-sq = 81·4	<0·0001
Low	48·4	45·3		
Medium	44·3	40·9		
High	7·3	13·7		
Education			chi-sq = 94·0	<0·0001
None	10·5	4·3		
Primary	24·5	20·0		
Secondary	40·9	46·5		
Tertiary	24·2	29·2		
Married or in a civil union	37·5	16·0	chi-sq = 297·3	<0·0001
Metropolitan Region	43·6	57·3	chi-sq = 110·6	<0·0001
Indigenous identity	8·8	12·3	chi-sq = 21·6	0·0060
Disability	8·7	4·3	chi-sq = 38·1	<0·0001
Childhood sexual abuse	11·1	18·4	chi-sq = 74·6	<0·0001
Individual Gender Index	26·9	23·0	t = -12·6	<0·0001
Own income	58·7	59·7	chi-sq = 0·6	0·66
Social participation	38·7	37·1	chi-sq = 1·6	0·44
Municipal Gender Index	27·0	26·8	t = -1·1	0·26
Municipal poverty rate	9·2	9·4	t = 0·6	0·57
Municipal crime rate	3 154	3 380	t = 2·7	0·0076

Weighted means or percentages. Survey-adjusted t-tests for continuous variables and design-adjusted Rao-Scott chi-square tests for categorical variables. t = survey-adjusted t statistic; chi-sq = design-adjusted Rao-Scott chi-square statistic; NPSH = non-partner sexual harassment; ENVIF = Encuesta Nacional de Violencia Intrafamiliar y en Otros Espacios (National Survey on Violence in the Family and Other Settings).

Supplementary Table 15. Survey-weighted multilevel model for IPSV

ENVIF 2017 and 2020

Variables	M0	M1	M2	M3
**Intercept**				
Intercept (baseline odds)	0·02 (0·02 to 0·03) ***	0·01 (0·00 to 0·04) ***	0·02 (0·01 to 0·02) ***	0·01 (0·00 to 0·03) ***
**Level 1 - Individual**				
Age group (ref: 15-18)				
19-25		0·91 (0·36 to 2·33)		0·95 (0·37 to 2·44)
26-35		0·52 (0·19 to 1·40)		0·53 (0·19 to 1·44)
36-45		0·57 (0·23 to 1·38)		0·58 (0·24 to 1·43)
46-55		0·52 (0·20 to 1·34)		0·52 (0·20 to 1·37)
56-65		0·57 (0·21 to 1·58)		0·57 (0·20 to 1·59)
66 or more		0·14 (0·02 to 0·83) *		0·12 (0·02 to 0·72) *
Socioeconomic level (ref: Low)				
Medium		0·85 (0·63 to 1·15)		0·91 (0·68 to 1·23)
High		1·35 (0·72 to 2·54)		1·38 (0·72 to 2·63)
Education (ref: None)				
Primary		1·33 (0·77 to 2·29)		1·26 (0·73 to 2·18)
Secondary		1·10 (0·63 to 1·94)		1·00 (0·57 to 1·76)
Tertiary		0·73 (0·37 to 1·44)		0·65 (0·33 to 1·29)
Married or in a civil union		1·07 (0·78 to 1·46)		1·10 (0·80 to 1·51)
Indigenous identity		1·18 (0·73 to 1·89)		1·14 (0·71 to 1·83)
Disability		1·42 (0·82 to 2·47)		1·35 (0·78 to 2·34)
Childhood sexual abuse		2·16 (1·42 to 3·27) ***		2·09 (1·35 to 3·23) ***
Individual Gender Index (z)		1·05 (0·87 to 1·27)		1·07 (0·88 to 1·31)
Own income		0·87 (0·63 to 1·19)		0·86 (0·63 to 1·19)
Social participation		1·13 (0·87 to 1·49)		1·08 (0·82 to 1·43)
**Level 1 - Relationship**				
Dominant relationship		6·20 (4·24 to 9·08) ***		6·17 (4·23 to 9·02) ***
Partner: violence in childhood		1·65 (1·14 to 2·39) **		1·67 (1·16 to 2·41) **
Partner: violent in other settings		2·46 (1·57 to 3·85) ***		2·36 (1·49 to 3·75) ***
**Level 2 - Municipality**				
Metropolitan Region			0·84 (0·55 to 1·29)	0·66 (0·44 to 0·99) *
Municipal Gender Index (z)			1·06 (0·90 to 1·26)	1·00 (0·82 to 1·21)
Municipal poverty rate (z)			1·06 (0·91 to 1·22)	0·97 (0·82 to 1·14)
Municipal crime rate (z)			1·03 (0·89 to 1·19)	1·05 (0·89 to 1·24)
**Level 2 - Survey wave**				
Survey wave: 2020 (ref: 2017)			1·72 (1·21 to 2·42) **	1·51 (1·04 to 2·19) *
**Model fit**				
n (women)	8 335	8 335	8 335	8 335
n (municipalities)				
AIC	1 896	1 641	1 888	1 641
BIC	1 910	1 803	1 937	1 838
Log-likelihood	-946·0	-797·7	-936·9	-792·5
VPC, municipality (%)	2·28	2·76	1·78	2·59

Variables	M0	M1	M2	M3
Median odds ratio	1·30	1·34	1·26	1·33

*OR (95% CI). Significance: *** p<0·001; ** p<0·01; * p<0·05. ENVIF = Encuesta Nacional de Violencia Intrafamiliar y en Otros Espacios. Survey-weighted two-level logistic regression estimated with WeMix using rescaled post-stratification weights at both levels. M0 = empty model (municipality random intercept only); M1 = M0 plus individual-level and relationship-level covariates; M2 = M0 plus contextual covariates (Metropolitan Region and municipal-level variables) and survey wave; M3 = full model with all covariates. OR = odds ratio; CI = confidence interval; ref = reference category; (z) = z-standardised variable (Individual Gender Index within survey wave; municipal covariates on the pooled analytic sample); AIC = Akaike information criterion; BIC = Bayesian information criterion; VPC = variance partition coefficient; IPSV = intimate partner sexual violence.*

Supplementary Table 16. Survey-weighted multilevel model for NPSA

ENVIF 2017 and 2020

Variables	M0	M1	M2	M3
**Intercept**				
Intercept (baseline odds)	0·02 (0·01 to 0·02) ***	0·03 (0·01 to 0·06) ***	0·01 (0·01 to 0·01) ***	0·01 (0·01 to 0·03) ***
**Level 1 - Individual**				
Age group (ref: 15-18)				
19-25		0·92 (0·53 to 1·60)		0·94 (0·54 to 1·63)
26-35		0·34 (0·17 to 0·67) **		0·35 (0·17 to 0·69) **
36-45		0·32 (0·16 to 0·63) **		0·33 (0·17 to 0·66) **
46-55		0·26 (0·14 to 0·48) ***		0·26 (0·14 to 0·49) ***
56-65		0·20 (0·10 to 0·40) ***		0·21 (0·10 to 0·42) ***
66 or more		0·06 (0·02 to 0·25) ***		0·05 (0·01 to 0·19) ***
Socioeconomic level (ref: Low)				
Medium		1·18 (0·86 to 1·62)		1·21 (0·85 to 1·71)
High		1·58 (0·74 to 3·37)		1·42 (0·63 to 3·20)
Education (ref: None)				
Primary		0·97 (0·54 to 1·72)		0·92 (0·51 to 1·66)
Secondary		1·12 (0·56 to 2·25)		1·03 (0·50 to 2·11)
Tertiary		0·83 (0·37 to 1·84)		0·68 (0·31 to 1·53)
Married or in a civil union		0·42 (0·27 to 0·64) ***		0·43 (0·28 to 0·67) ***
Indigenous identity		1·19 (0·81 to 1·76)		1·23 (0·84 to 1·82)
Disability		1·53 (0·95 to 2·48)		1·43 (0·89 to 2·30)
Childhood sexual abuse		3·29 (2·19 to 4·93) ***		2·92 (1·98 to 4·31) ***
Individual Gender Index (z)		0·86 (0·69 to 1·07)		0·82 (0·64 to 1·06)
Own income		1·11 (0·81 to 1·51)		1·09 (0·80 to 1·48)
Social participation		1·49 (1·12 to 1·99) **		1·53 (1·15 to 2·03) **
**Level 2 - Municipality**				
Metropolitan Region			2·70 (1·90 to 3·84) ***	2·96 (2·03 to 4·32) ***
Municipal Gender Index (z)			1·06 (0·85 to 1·32)	1·14 (0·89 to 1·45)
Municipal poverty rate (z)			1·07 (0·87 to 1·31)	1·04 (0·85 to 1·28)
Municipal crime rate (z)			1·08 (0·94 to 1·23)	1·08 (0·95 to 1·23)
**Level 2 - Survey wave**				
Survey wave: 2020 (ref: 2017)			2·00 (1·35 to 2·95) ***	1·93 (1·29 to 2·88) **
**Model fit**				
n (women)	13 128	13 128	13 128	13 128
n (municipalities)				
AIC	2 383	2 189	2 329	2 133
BIC	2 398	2 339	2 381	2 320
Log-likelihood	-1189·5	-1074·5	-1157·5	-1041·5
VPC, municipality (%)	7·63	7·40	5·26	5·23

Variables	M0	M1	M2	M3
Median odds ratio	1·64	1·63	1·50	1·50

*OR (95% CI). Significance: *** p<0·001; ** p<0·01; * p<0·05. ENVIF = Encuesta Nacional de Violencia Intrafamiliar y en Otros Espacios. Survey-weighted two-level logistic regression estimated with WeMix using rescaled post-stratification weights at both levels. M0 = empty model (municipality random intercept only); M1 = M0 plus individual-level covariates; M2 = M0 plus contextual covariates (Metropolitan Region and municipal-level variables) and survey wave; M3 = full model with all covariates. OR = odds ratio; CI = confidence interval; ref = reference category; (z) = z-standardised variable (Individual Gender Index within survey wave; municipal covariates on the pooled analytic sample); AIC = Akaike information criterion; BIC = Bayesian information criterion; VPC = variance partition coefficient; NPSA = non-partner sexual assault.*

Supplementary Table 17. Survey-weighted multilevel model for NPSH

ENVIF 2017 and 2020

Variables	M0	M1	M2	M3
**Intercept**				
Intercept (baseline odds)	0·11 (0·09 to 0·12) ***	0·32 (0·21 to 0·50) ***	0·05 (0·04 to 0·06) ***	0·16 (0·10 to 0·25) ***
**Level 1 - Individual**				
Age group (ref: 15-18)				
19-25		0·57 (0·43 to 0·76) ***		0·59 (0·44 to 0·79) ***
26-35		0·24 (0·17 to 0·33) ***		0·25 (0·17 to 0·35) ***
36-45		0·14 (0·10 to 0·19) ***		0·14 (0·10 to 0·20) ***
46-55		0·11 (0·08 to 0·16) ***		0·11 (0·08 to 0·16) ***
56-65		0·06 (0·04 to 0·09) ***		0·06 (0·04 to 0·09) ***
66 or more		0·04 (0·02 to 0·08) ***		0·03 (0·01 to 0·06) ***
Socioeconomic level (ref: Low)				
Medium		0·82 (0·69 to 0·97) *		0·90 (0·76 to 1·08)
High		1·09 (0·77 to 1·56)		1·05 (0·74 to 1·47)
Education (ref: None)				
Primary		0·68 (0·46 to 1·00) *		0·62 (0·41 to 0·93) *
Secondary		1·02 (0·67 to 1·54)		0·91 (0·60 to 1·36)
Tertiary		1·29 (0·84 to 1·99)		1·04 (0·68 to 1·60)
Married or in a civil union		0·68 (0·58 to 0·81) ***		0·73 (0·62 to 0·86) ***
Indigenous identity		1·09 (0·85 to 1·40)		1·08 (0·86 to 1·34)
Disability		1·26 (0·91 to 1·74)		1·13 (0·81 to 1·58)
Childhood sexual abuse		2·26 (1·90 to 2·69) ***		2·00 (1·69 to 2·36) ***
Individual Gender Index (z)		0·66 (0·60 to 0·73) ***		0·66 (0·60 to 0·73) ***
Own income		1·22 (1·03 to 1·45) *		1·17 (0·98 to 1·39)
Social participation		1·38 (1·15 to 1·67) ***		1·32 (1·08 to 1·61) **
**Level 2 - Municipality**				
Metropolitan Region			1·95 (1·53 to 2·50) ***	2·21 (1·72 to 2·84) ***
Municipal Gender Index (z)			0·95 (0·83 to 1·10)	1·13 (0·98 to 1·31)
Municipal poverty rate (z)			1·14 (0·98 to 1·31)	1·13 (0·97 to 1·31)
Municipal crime rate (z)			1·15 (1·03 to 1·28) **	1·15 (1·03 to 1·29) *
**Level 2 - Survey wave**				
Survey wave: 2020 (ref: 2017)			2·65 (2·01 to 3·50) ***	2·66 (1·98 to 3·57) ***
**Model fit**				
n (women)	13 128	13 128	13 128	13 128
n (municipalities)				
AIC	8 195	7 043	7 922	6 759
BIC	8 210	7 193	7 974	6 946
Log-likelihood	-4095·5	-3501·6	-3953·9	-3354·5
VPC, municipality (%)	8·67	8·43	5·17	5·23

Variables	M0	M1	M2	M3
Median odds ratio	1·70	1·69	1·50	1·50

*OR (95% CI). Significance: *** p<0·001; ** p<0·01; * p<0·05. ENVIF = Encuesta Nacional de Violencia Intrafamiliar y en Otros Espacios. Survey-weighted two-level logistic regression estimated with WeMix using rescaled post-stratification weights at both levels. M0 = empty model (municipality random intercept only); M1 = M0 plus individual-level covariates; M2 = M0 plus contextual covariates (Metropolitan Region and municipal-level variables) and survey wave; M3 = full model with all covariates. OR = odds ratio; CI = confidence interval; ref = reference category; (z) = z-standardised variable (Individual Gender Index within survey wave; municipal covariates on the pooled analytic sample); AIC = Akaike information criterion; BIC = Bayesian information criterion; VPC = variance partition coefficient; NPSH = non-partner sexual harassment. Confidence limits are shown to two decimal places; a limit displayed as 1·00 may still exclude the null value, as indicated by the significance marker.*

Supplementary Table 18. Single-level logistic model for IPSV

Fully adjusted model, ENVIF 2017 and 2020

Variables	M3
**Intercept**	
Intercept (baseline odds)	0·02 (0·01 to 0·04) ***
**Level 1 - Individual**	
Age group (ref: 15-18)	
19-25	0·84 (0·36 to 1·97)
26-35	0·46 (0·20 to 1·07)
36-45	0·51 (0·22 to 1·20)
46-55	0·45 (0·19 to 1·06)
56-65	0·47 (0·20 to 1·13)
66 or more	0·09 (0·02 to 0·46) **
Socioeconomic level (ref: Low)	
Medium	0·90 (0·65 to 1·26)
High	1·35 (0·67 to 2·69)
Education (ref: None)	
Primary	1·15 (0·67 to 1·99)
Secondary	0·94 (0·54 to 1·64)
Tertiary	0·56 (0·28 to 1·11)
Married or in a civil union	1·21 (0·85 to 1·72)
Indigenous identity	1·08 (0·71 to 1·62)
Disability	1·37 (0·85 to 2·22)
Childhood sexual abuse	2·05 (1·45 to 2·89) ***
Individual Gender Index (z)	1·04 (0·88 to 1·24)
Own income	0·84 (0·61 to 1·14)
Social participation	1·07 (0·79 to 1·45)
**Level 1 - Relationship**	
Dominant relationship	6·40 (4·68 to 8·76) ***
Partner: violence in childhood	1·60 (1·17 to 2·18) **
Partner: violent in other settings	2·79 (1·75 to 4·43) ***
**Level 2 - Municipality**	
Metropolitan Region	0·61 (0·38 to 0·96) *
Municipal Gender Index (z)	1·00 (0·83 to 1·21)
Municipal poverty rate (z)	0·98 (0·82 to 1·16)
Municipal crime rate (z)	1·02 (0·86 to 1·21)
**Level 2 - Survey wave**	
Survey wave: 2020 (ref: 2017)	1·41 (1·02 to 1·95) *
**Model fit**	
n (women)	8 335
AIC	1 623
BIC	1 813
Log-likelihood	-784·5

*OR (95% CI). Significance: *** p<0·001; ** p<0·01; * p<0·05. ENVIF = Encuesta Nacional de Violencia Intrafamiliar y en Otros Espacios. Single-level logistic regression without a municipality random intercept. M3 = fully adjusted model with all covariates. OR = odds ratio; CI = confidence interval; ref = reference category; (z) = z-standardised variable (Individual Gender Index within survey wave; municipal covariates on the pooled analytic sample); AIC = Akaike information criterion; BIC = Bayesian information criterion; IPSV = intimate partner sexual violence.*

Supplementary Table 19. Single-level logistic model for NPSA

Fully adjusted model, ENVIF 2017 and 2020

Variables	M3
**Intercept**	
Intercept (baseline odds)	0·02 (0·01 to 0·04) ***
**Level 1 - Individual**	
Age group (ref: 15-18)	
19-25	0·85 (0·48 to 1·49)
26-35	0·32 (0·17 to 0·59) ***
36-45	0·30 (0·16 to 0·57) ***
46-55	0·25 (0·13 to 0·48) ***
56-65	0·21 (0·11 to 0·40) ***
66 or more	0·04 (0·01 to 0·19) ***
Socioeconomic level (ref: Low)	
Medium	1·16 (0·84 to 1·59)
High	1·31 (0·72 to 2·36)
Education (ref: None)	
Primary	0·82 (0·41 to 1·64)
Secondary	1·01 (0·52 to 1·95)
Tertiary	0·69 (0·33 to 1·43)
Married or in a civil union	0·47 (0·31 to 0·72) ***
Indigenous identity	1·21 (0·83 to 1·77)
Disability	1·27 (0·75 to 2·16)
Childhood sexual abuse	3·02 (2·19 to 4·17) ***
Individual Gender Index (z)	0·85 (0·71 to 1·01)
Own income	1·16 (0·84 to 1·60)
Social participation	1·50 (1·12 to 2·01) **
**Level 2 - Municipality**	
Metropolitan Region	2·85 (2·03 to 3·99) ***
Municipal Gender Index (z)	1·10 (0·91 to 1·33)
Municipal poverty rate (z)	1·03 (0·85 to 1·24)
Municipal crime rate (z)	1·09 (0·95 to 1·26)
**Level 2 - Survey wave**	
Survey wave: 2020 (ref: 2017)	2·14 (1·51 to 3·01) ***
**Model fit**	
n (women)	13 128
AIC	1 863
BIC	2 043
Log-likelihood	-907·6

*OR (95% CI). Significance: *** p<0·001; ** p<0·01; * p<0·05. ENVIF = Encuesta Nacional de Violencia Intrafamiliar y en Otros Espacios. Single-level logistic regression without a municipality random intercept. M3 = fully adjusted model with all covariates. OR = odds ratio; CI = confidence interval; ref = reference category; (z) = z-standardised variable (Individual Gender Index within survey wave; municipal covariates on the pooled analytic sample); AIC = Akaike information criterion; BIC = Bayesian information criterion; NPSA = non-partner sexual assault.*

Supplementary Table 20. Single-level logistic model for NPSH

Fully adjusted model, ENVIF 2017 and 2020

Variables	M3
**Intercept**	
Intercept (baseline odds)	0·15 (0·10 to 0·22) ***
**Level 1 - Individual**	
Age group (ref: 15-18)	
19-25	0·60 (0·45 to 0·80) ***
26-35	0·25 (0·19 to 0·34) ***
36-45	0·14 (0·10 to 0·20) ***
46-55	0·12 (0·08 to 0·16) ***
56-65	0·06 (0·05 to 0·09) ***
66 or more	0·03 (0·01 to 0·05) ***
Socioeconomic level (ref: Low)	
Medium	0·91 (0·78 to 1·07)
High	1·04 (0·80 to 1·36)
Education (ref: None)	
Primary	0·71 (0·49 to 1·03)
Secondary	1·01 (0·71 to 1·43)
Tertiary	1·18 (0·82 to 1·71)
Married or in a civil union	0·77 (0·64 to 0·92) **
Indigenous identity	1·08 (0·89 to 1·30)
Disability	1·21 (0·90 to 1·62)
Childhood sexual abuse	2·09 (1·75 to 2·50) ***
Individual Gender Index (z)	0·68 (0·62 to 0·74) ***
Own income	1·18 (1·01 to 1·38) *
Social participation	1·24 (1·07 to 1·42) **
**Level 2 - Municipality**	
Metropolitan Region	2·06 (1·72 to 2·46) ***
Municipal Gender Index (z)	1·10 (1·00 to 1·20)
Municipal poverty rate (z)	1·10 (1·01 to 1·20) *
Municipal crime rate (z)	1·14 (1·07 to 1·22) ***
**Level 2 - Survey wave**	
Survey wave: 2020 (ref: 2017)	2·71 (2·30 to 3·20) ***
**Model fit**	
n (women)	13 128
AIC	5 942
BIC	6 121
Log-likelihood	-2946·9

*OR (95% CI). Significance: *** p<0·001; ** p<0·01; * p<0·05. ENVIF = Encuesta Nacional de Violencia Intrafamiliar y en Otros Espacios. Single-level logistic regression without a municipality random intercept. M3 = fully adjusted model with all covariates. OR = odds ratio; CI = confidence interval; ref = reference category; (z) = z-standardised variable (Individual Gender Index within survey wave; municipal covariates on the pooled analytic sample); AIC = Akaike information criterion; BIC = Bayesian information criterion; NPSH = non-partner sexual harassment.*

Supplementary Table 21. Fully adjusted models estimated separately by wave

ENVIF 2017 and ENVIF 2020

Variables	IPSV 2017	IPSV 2020	NPSA 2017	NPSA 2020	NPSH 2017	NPSH 2020
Age group: 19-25	0·64 (0·18 to 2·34)	1·03 (0·34 to 3·17)	1·67 (0·55 to 5·09)	0·65 (0·33 to 1·27)	0·65 (0·38 to 1·12)	0·58 (0·41 to 0·83) **
Age group: 26-35	0·38 (0·10 to 1·35)	0·53 (0·17 to 1·65)	0·47 (0·14 to 1·64)	0·26 (0·13 to 0·55) ***	0·25 (0·14 to 0·45) ***	0·25 (0·17 to 0·36) ***
Age group: 36-45	0·34 (0·09 to 1·27)	0·67 (0·22 to 2·07)	0·49 (0·14 to 1·77)	0·23 (0·11 to 0·50) ***	0·15 (0·08 to 0·29) ***	0·14 (0·09 to 0·20) ***
Age group: 46-55	0·26 (0·07 to 1·00) *	0·61 (0·20 to 1·89)	0·44 (0·12 to 1·62)	0·19 (0·09 to 0·43) ***	0·14 (0·07 to 0·25) ***	0·11 (0·07 to 0·16) ***
Age group: 56-65	0·42 (0·11 to 1·59)	0·47 (0·15 to 1·51)	0·22 (0·05 to 0·95) *	0·20 (0·09 to 0·42) ***	0·06 (0·03 to 0·12) ***	0·06 (0·04 to 0·09) ***
Age group: 66 or more		0·11 (0·02 to 0·66) *		0·04 (0·01 to 0·18) ***		0·03 (0·01 to 0·06) ***
Socioeconomic level: Medium	0·89 (0·53 to 1·50)	0·95 (0·62 to 1·47)	0·73 (0·40 to 1·33)	1·43 (0·97 to 2·09)	0·93 (0·69 to 1·27)	0·91 (0·75 to 1·10)
Socioeconomic level: High	0·70 (0·15 to 3·28)	1·84 (0·83 to 4·09)	0·73 (0·19 to 2·72)	1·71 (0·87 to 3·36)	1·20 (0·64 to 2·22)	1·00 (0·73 to 1·37)
Education: Primary	1·55 (0·66 to 3·66)	0·91 (0·45 to 1·87)	0·81 (0·27 to 2·46)	0·90 (0·37 to 2·19)	0·80 (0·43 to 1·52)	0·68 (0·43 to 1·09)
Education: Secondary	0·88 (0·35 to 2·18)	0·89 (0·43 to 1·84)	0·75 (0·26 to 2·19)	1·25 (0·53 to 2·92)	0·86 (0·47 to 1·59)	1·09 (0·70 to 1·70)
Education: Tertiary	0·61 (0·19 to 1·94)	0·47 (0·19 to 1·13)	0·78 (0·23 to 2·62)	0·77 (0·30 to 1·97)	0·69 (0·35 to 1·35)	1·37 (0·86 to 2·18)
Married or in a civil union	1·12 (0·62 to 2·03)	1·30 (0·84 to 2·02)	0·48 (0·22 to 1·04)	0·45 (0·27 to 0·74) **	0·59 (0·41 to 0·86) **	0·82 (0·66 to 1·01)
Metropolitan Region	0·72 (0·38 to 1·39)	0·53 (0·27 to 1·06)	6·15 (3·41 to 11·11) ***	1·82 (1·11 to 3·01) *	4·06 (2·32 to 7·12) ***	1·70 (1·26 to 2·31) ***
Indigenous identity	0·85 (0·41 to 1·79)	1·14 (0·69 to 1·88)	0·52 (0·18 to 1·47)	1·48 (0·98 to 2·24)	0·90 (0·58 to 1·40)	1·18 (0·94 to 1·48)
Disability	2·27 (0·99 to 5·22)	1·14 (0·63 to 2·06)	1·06 (0·30 to 3·73)	1·24 (0·68 to 2·25)	1·36 (0·72 to 2·58)	1·12 (0·80 to 1·56)
Childhood sexual abuse	1·74 (0·90 to 3·35)	2·18 (1·45 to 3·29) ***	2·75 (1·37 to 5·50) **	3·11 (2·14 to 4·51) ***	2·11 (1·42 to 3·13) ***	2·04 (1·66 to 2·51) ***
Individual Gender Index (z)	1·05 (0·80 to 1·38)	1·07 (0·86 to 1·32)	0·54 (0·37 to 0·79) **	1·00 (0·82 to 1·23)	0·71 (0·59 to 0·86) ***	0·68 (0·62 to 0·76) ***
Own income	1·18 (0·70 to 2·00)	0·69 (0·47 to 1·01)	0·86 (0·47 to 1·57)	1·35 (0·92 to 2·01)	1·41 (1·03 to 1·93) *	1·12 (0·92 to 1·35)
Social participation	1·03 (0·62 to 1·72)	1·06 (0·72 to 1·55)	1·45 (0·81 to 2·58)	1·44 (1·02 to 2·02) *	1·36 (1·02 to 1·82) *	1·21 (1·03 to 1·44) *
Dominant relationship	5·33 (3·19 to 8·93) ***	7·26 (4·87 to 10·82) ***				
Partner: violence in childhood	1·84 (1·10 to 3·07) *	1·51 (1·02 to 2·24) *				
Partner: violent in other settings	6·56 (3·33 to 12·90) ***	1·69 (0·90 to 3·17)				
Municipal Gender Index (z)	1·14 (0·90 to 1·44)	0·73 (0·51 to 1·05)	1·37 (1·06 to 1·78) *	0·96 (0·67 to 1·38)	1·16 (0·98 to 1·39)	0·98 (0·80 to 1·20)
Municipal poverty rate (z)	0·81 (0·63 to 1·04)	1·29 (0·97 to 1·71)	0·89 (0·65 to 1·23)	1·16 (0·87 to 1·55)	1·25 (1·02 to 1·53) *	1·03 (0·86 to 1·22)
Municipal crime rate (z)	1·04 (0·82 to 1·32)	0·92 (0·72 to 1·18)	0·92 (0·72 to 1·17)	1·22 (0·98 to 1·50)	1·21 (0·98 to 1·49)	1·08 (0·95 to 1·23)

*OR (95% CI). Significance: *** p<0·001; ** p<0·01; * p<0·05. ENVIF = Encuesta Nacional de Violencia Intrafamiliar y en Otros Espacios. The survey-wave indicator is not estimable within a single wave and is therefore omitted from these models. OR = odds ratio; CI = confidence interval; ref = reference category; (z) = z-standardised variable (Individual Gender Index within survey wave; municipal covariates on the pooled analytic sample); IPSV = intimate partner sexual violence; NPSA = non-partner sexual assault; NPSH = non-partner sexual harassment.*

Supplementary Table 22. Likelihood-ratio tests for survey-wave interactions

Fully adjusted models with and without wave interactions

Outcome	Interaction terms	Chi-square	df	p-value	AIC additive	AIC interaction
IPSV	Dominant relationship, childhood sexual abuse, Metropolitan Region, age group	6·60	8	0·58	1 625	1 634
NPSA	Childhood sexual abuse, Metropolitan Region, age group	15·43	7	0·031	1 862	1 861
NPSH	Childhood sexual abuse, Metropolitan Region, age group	18·86	7	0·0086	5 909	5 905

Each fully adjusted model was refitted adding interactions between survey wave and the strongest determinants, then compared with the additive model by likelihood-ratio test. A non-significant test supports pooling the two waves. df = degrees of freedom; AIC = Akaike information criterion; IPSV = intimate partner sexual violence; NPSA = non-partner sexual assault; NPSH = non-partner sexual harassment.

Supplementary Table 23. IPSV fully adjusted model restricted to the 2020 wave

Addresses the difference in the response format of the partner items between rounds and in the administration of the IPSV battery in 2017

Variables	M3 pooled	M3, 2020 only
Age group: 19-25	0·84 (0·36 to 1·97)	1·03 (0·34 to 3·17)
Age group: 26-35	0·46 (0·20 to 1·07)	0·53 (0·17 to 1·65)
Age group: 36-45	0·51 (0·22 to 1·20)	0·67 (0·22 to 2·07)
Age group: 46-55	0·45 (0·19 to 1·06)	0·61 (0·20 to 1·89)
Age group: 56-65	0·47 (0·20 to 1·13)	0·47 (0·15 to 1·51)
Age group: 66 or more	0·09 (0·02 to 0·46) **	0·11 (0·02 to 0·66) *
Socioeconomic level: Medium	0·90 (0·65 to 1·26)	0·95 (0·62 to 1·47)
Socioeconomic level: High	1·35 (0·67 to 2·69)	1·84 (0·83 to 4·09)
Education: Primary	1·15 (0·67 to 1·99)	0·91 (0·45 to 1·87)
Education: Secondary	0·94 (0·54 to 1·64)	0·89 (0·43 to 1·84)
Education: Tertiary	0·56 (0·28 to 1·11)	0·47 (0·19 to 1·13)
Married or in a civil union	1·21 (0·85 to 1·72)	1·30 (0·84 to 2·02)
Metropolitan Region	0·61 (0·38 to 0·96) *	0·53 (0·27 to 1·06)
Indigenous identity	1·08 (0·71 to 1·62)	1·14 (0·69 to 1·88)
Disability	1·37 (0·85 to 2·23)	1·14 (0·63 to 2·06)
Childhood sexual abuse	2·05 (1·45 to 2·89) ***	2·18 (1·45 to 3·29) ***
Individual Gender Index (z)	1·04 (0·88 to 1·24)	1·07 (0·86 to 1·32)
Own income	0·84 (0·61 to 1·14)	0·69 (0·47 to 1·01)
Social participation	1·07 (0·79 to 1·45)	1·06 (0·72 to 1·55)
Dominant relationship	6·40 (4·68 to 8·76) ***	7·26 (4·87 to 10·82) ***
Partner: violence in childhood	1·60 (1·17 to 2·18) **	1·51 (1·02 to 2·24) *
Partner: violent in other settings	2·79 (1·75 to 4·43) ***	1·69 (0·90 to 3·17)
Municipal Gender Index (z)	1·00 (0·83 to 1·21)	0·73 (0·51 to 1·05)
Municipal poverty rate (z)	0·98 (0·82 to 1·16)	1·29 (0·97 to 1·71)
Municipal crime rate (z)	1·02 (0·86 to 1·21)	0·92 (0·72 to 1·18)
Survey wave: 2020 (ref: 2017)	1·41 (1·02 to 1·95) *	
n (women)	8 335	4 318
n (municipalities)	241	148
AIC	1 625	995
BIC	1 822	1 167
VPC (%)	0·00	0·00

*OR (95% CI). Significance: *** p<0·001; ** p<0·01; * p<0·05. ENVIF = Encuesta Nacional de Violencia Intrafamiliar y en Otros Espacios. M3 = fully adjusted model with all covariates. OR = odds ratio; CI = confidence interval; ref = reference category; (z) = z-standardised variable (Individual Gender Index within survey wave; municipal covariates on the pooled analytic sample); AIC = Akaike information criterion; BIC = Bayesian information criterion; VPC = variance partition coefficient; IPSV = intimate partner sexual violence.*

Supplementary Table 24a. Events per variable in the fully adjusted models

Outcome	n (women)	Outcome events	Estimated parameters	Events per variable
IPSV	8 335	199	26	7·65
NPSA	13 128	200	23	8·70
NPSH	13 128	1 028	23	44·70

Unweighted counts of outcome events entering each model, and the ratio of events to estimated fixed-effect parameters (excluding the intercept). IPSV = intimate partner sexual violence; NPSA = non-partner sexual assault; NPSH = non-partner sexual harassment.

Supplementary Table 24b. IPSV events by age group

IPSV analytic sample

Age group	Non-victims	Victims
15-18	137	9
19-25	754	33
26-35	1 776	35
36-45	1 694	38
46-55	1 758	40
56-65	1 691	42
66 or more	326	2

Sparse cells in the oldest age groups explain the wide confidence intervals for those categories in table 3; a Firth penalised-likelihood sensitivity analysis is reported in Supplementary Table 24c.

Supplementary Table 24c. IPSV fully adjusted model: Firth penalised likelihood

Variables	Maximum likelihood	Firth penalised
Age group: 19-25	0·84 (0·36 to 1·97)	0·82 (0·37 to 1·97)
Age group: 26-35	0·46 (0·20 to 1·07)	0·45 (0·20 to 1·08)
Age group: 36-45	0·51 (0·22 to 1·20)	0·50 (0·22 to 1·21)
Age group: 46-55	0·45 (0·19 to 1·06)	0·44 (0·20 to 1·07)
Age group: 56-65	0·47 (0·20 to 1·13)	0·46 (0·20 to 1·14)
Age group: 66 or more	0·09 (0·02 to 0·46)	0·11 (0·02 to 0·44)
Socioeconomic level: Medium	0·90 (0·65 to 1·26)	0·90 (0·65 to 1·25)
Socioeconomic level: High	1·35 (0·67 to 2·69)	1·38 (0·67 to 2·64)
Education: Primary	1·15 (0·67 to 1·99)	1·14 (0·68 to 1·98)
Education: Secondary	0·94 (0·54 to 1·64)	0·92 (0·54 to 1·64)
Education: Tertiary	0·56 (0·28 to 1·11)	0·56 (0·28 to 1·11)
Married or in a civil union	1·21 (0·85 to 1·72)	1·20 (0·85 to 1·71)
Metropolitan Region	0·61 (0·38 to 0·96)	0·62 (0·39 to 0·96)
Indigenous identity	1·08 (0·71 to 1·62)	1·09 (0·72 to 1·62)
Disability	1·37 (0·85 to 2·23)	1·39 (0·84 to 2·20)
Childhood sexual abuse	2·05 (1·45 to 2·89)	2·04 (1·44 to 2·85)
Individual Gender Index (z)	1·04 (0·88 to 1·24)	1·04 (0·88 to 1·23)
Own income	0·84 (0·61 to 1·14)	0·84 (0·62 to 1·14)
Social participation	1·07 (0·79 to 1·45)	1·07 (0·79 to 1·45)
Dominant relationship	6·40 (4·68 to 8·76)	6·30 (4·62 to 8·64)
Partner: violence in childhood	1·60 (1·17 to 2·18)	1·59 (1·17 to 2·17)
Partner: violent in other settings	2·79 (1·75 to 4·43)	2·77 (1·73 to 4·33)
Municipal Gender Index (z)	1·00 (0·83 to 1·21)	1·00 (0·84 to 1·21)
Municipal poverty rate (z)	0·98 (0·82 to 1·16)	0·98 (0·83 to 1·16)
Municipal crime rate (z)	1·02 (0·86 to 1·21)	1·02 (0·86 to 1·20)

Variables	Maximum likelihood	Firth penalised
Survey wave: 2020 (ref: 2017)	1·41 (1·02 to 1·95)	1·39 (1·01 to 1·92)

OR (95% CI). The penalised model is fitted without a municipality random intercept, which is defensible here because the estimated between-municipality variance for IPSV is at its boundary (Supplementary Table 8a). Penalised intervals are profile-likelihood intervals. OR = odds ratio; CI = confidence interval; ref = reference category; (z) = z-standardised variable (Individual Gender Index within survey wave; municipal covariates on the pooled analytic sample); IPSV = intimate partner sexual violence.

Supplementary Table 25a. IPSV prevalence under the two item definitions

Women with a partner in the last 12 months

Definition	2017	2020	Pooled
IPSV, 5 common items	2·1 (1·5 to 2·8)	3·5 (2·8 to 4·2)	2·8 (2·4 to 3·3)
IPSV, 8 items (2020 battery)	2·1 (1·5 to 2·8)	4·1 (3·3 to 4·9)	3·2 (2·7 to 3·7)

The 2017 instrument comprises five items. The 2020 instrument added three (forced viewing of pornography, forced unprotected sex, and forced recording of sexual content). The primary analysis uses the five items common to both waves, so the outcome is measurement-invariant across the pooled sample. The eight-item definition is shown for comparison; in 2017 the two definitions coincide by construction. IPSV = intimate partner sexual violence.

Supplementary Table 25b. Fully adjusted IPSV model under the two item definitions

Same analytic sample, ENVIF 2017 and 2020

Variables	M3, 5 common items	M3, 8 items
Age group: 19-25	0·84 (0·36 to 1·97)	0·81 (0·36 to 1·81)
Age group: 26-35	0·46 (0·20 to 1·07)	0·43 (0·19 to 0·97) *
Age group: 36-45	0·51 (0·22 to 1·20)	0·47 (0·21 to 1·07)
Age group: 46-55	0·45 (0·19 to 1·06)	0·45 (0·20 to 1·02)
Age group: 56-65	0·47 (0·20 to 1·13)	0·44 (0·19 to 1·02)
Age group: 66 or more	0·09 (0·02 to 0·46) **	0·08 (0·02 to 0·39) **
Socioeconomic level: Medium	0·90 (0·65 to 1·26)	0·87 (0·63 to 1·20)
Socioeconomic level: High	1·35 (0·67 to 2·69)	1·22 (0·61 to 2·43)
Education: Primary	1·15 (0·67 to 1·99)	1·21 (0·71 to 2·09)
Education: Secondary	0·94 (0·54 to 1·64)	1·03 (0·59 to 1·80)
Education: Tertiary	0·56 (0·28 to 1·11)	0·64 (0·32 to 1·25)
Married or in a civil union	1·21 (0·85 to 1·72)	1·13 (0·81 to 1·60)
Metropolitan Region	0·61 (0·38 to 0·96) *	0·61 (0·39 to 0·96) *
Indigenous identity	1·08 (0·71 to 1·62)	1·12 (0·75 to 1·66)
Disability	1·37 (0·85 to 2·23)	1·57 (1·00 to 2·48)
Childhood sexual abuse	2·05 (1·45 to 2·89) ***	2·11 (1·51 to 2·95) ***
Individual Gender Index (z)	1·04 (0·88 to 1·24)	1·04 (0·88 to 1·23)
Own income	0·84 (0·61 to 1·14)	0·80 (0·59 to 1·08)
Social participation	1·07 (0·79 to 1·45)	1·05 (0·78 to 1·41)
Dominant relationship	6·40 (4·68 to 8·76) ***	6·11 (4·50 to 8·29) ***
Partner: violence in childhood	1·60 (1·17 to 2·18) **	1·66 (1·23 to 2·25) **
Partner: violent in other settings	2·79 (1·75 to 4·43) ***	2·68 (1·69 to 4·24) ***
Municipal Gender Index (z)	1·00 (0·83 to 1·21)	0·99 (0·82 to 1·18)
Municipal poverty rate (z)	0·98 (0·82 to 1·16)	1·00 (0·84 to 1·18)
Municipal crime rate (z)	1·02 (0·86 to 1·21)	1·02 (0·86 to 1·21)
Survey wave: 2020 (ref: 2017)	1·41 (1·02 to 1·95) *	1·53 (1·11 to 2·10) **

*OR (95% CI). Significance: *** p<0·001; ** p<0·01; * p<0·05. ENVIF = Encuesta Nacional de Violencia Intrafamiliar y en Otros Espacios. M3 = fully adjusted model with all covariates. OR = odds ratio; CI = confidence interval; ref = reference category; (z) = z-standardised variable (Individual Gender Index within survey wave; municipal covariates on the pooled analytic sample); IPSV = intimate partner sexual violence.*

Supplementary figures

Supplementary Figure 1. Participant flow, ENVIF 2017 and 2020

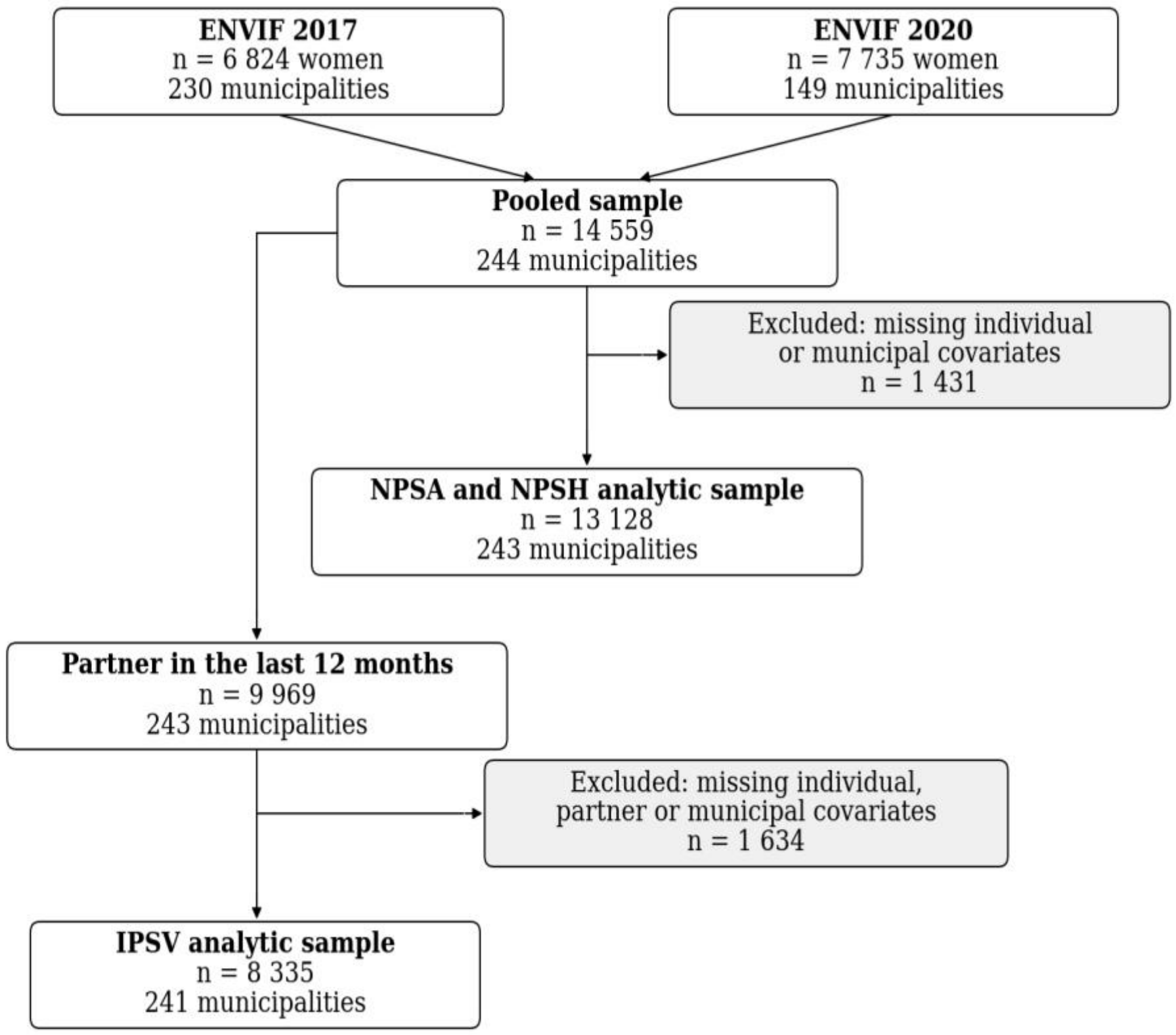

Derivation of the three analytic samples from the pooled 2017 and 2020 rounds of the Chilean National Survey on Violence in the Family and Other Settings (ENVIF): the full pooled sample (n = 14 559); the analytic sample for non-partner sexual assault (NPSA) and non-partner sexual harassment (NPSH) after exclusion of women with missing individual-level or municipal covariates (n = 13 128); women reporting a partner in the previous 12 months (n = 9 969); and the analytic sample for intimate partner sexual violence (IPSV) after further exclusion of women with missing individual-level, relationship-level or municipal covariates (n = 8 335). Numbers are unweighted counts of women and of municipalities.

Supplementary Figure 2. Past-12-month prevalence by age group and region. Weighted 12 month prevalence (%) with 95% confidence intervals (CI) of intimate partner sexual violence (IPSV), non-partner

sexual assault (NPSA) and non-partner sexual harassment (NPSH) by (a) age group and (b) region, pooled 2017 and 2020 rounds of the Chilean National Survey on Violence in the Family and Other Settings (ENVIF). Estimates use ENVIF post-stratification weights with clustering at the primary sampling unit. IPSV denominator: women with a partner in the previous 12 months; NPSA and NPSH denominator: full sample.

Supplementary Figure 2a

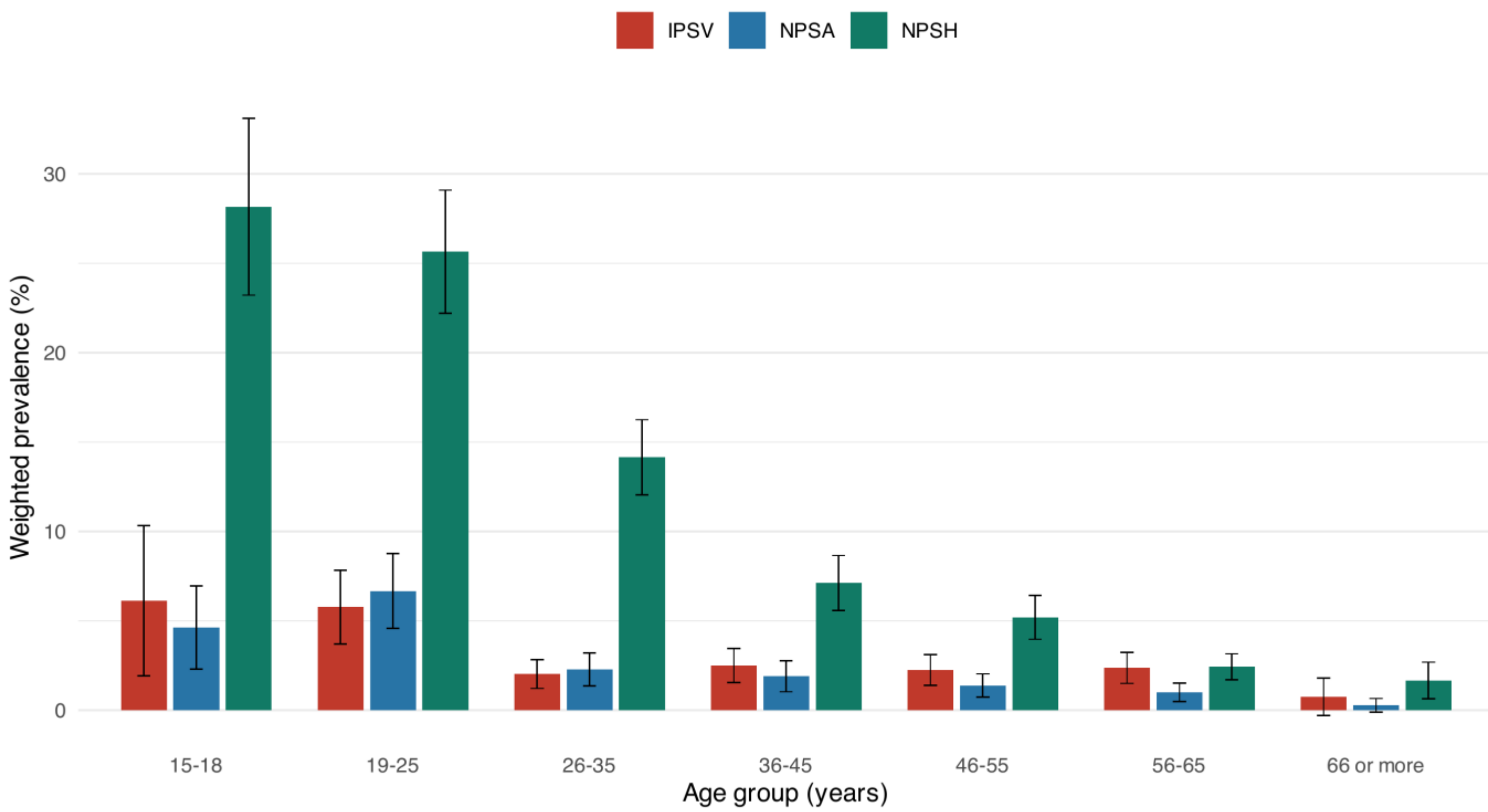

Weighted prevalence with 95% confidence intervals, ENVIF 2017 and 2020 pooled.

Supplementary Figure 2b

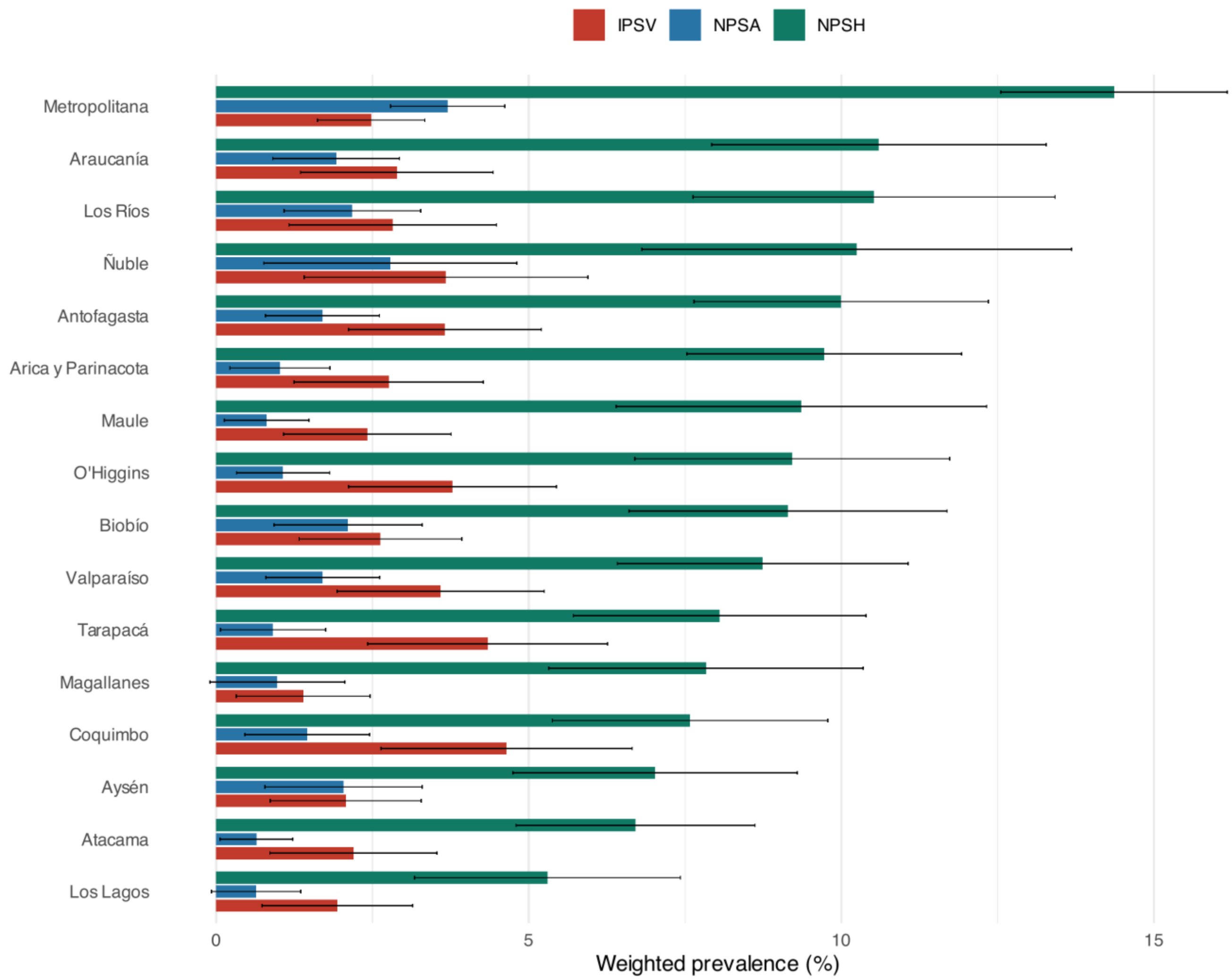

Weighted prevalence with 95% confidence intervals, ENVIF 2017 and 2020 pooled.
